# The Expression Profile of mRNA and tRNA Genes in Splenocytes and Neutrophils after In Vivo Delivery of Antitumor Short Hairpin RNA of Indoleamine 2,3- Dioxygenase

**DOI:** 10.3390/ijms21186703

**Published:** 2020-09-13

**Authors:** Ming-Shyan Huang, Ya-Ling Hsu, I-Jeng Yeh, Kuan-Ting Liu, Meng-Chi Yen

**Affiliations:** 1 Department of Internal Medicine, E-DA Cancer Hospital, Kaohsiung 840, Taiwan; shyan310@gmail.com; 2 School of Medicine, I-Shou University, Kaohsiung 840, Taiwan; 3 Graduate Institute of Medicine, College of Medicine, Kaohsiung Medical University, Kaohsiung 807, Taiwan; hsuyl326@gmail.com; 4 Department of Medical Research, Kaohsiung Medical University Hospital, Kaohsiung 807, Taiwan; 5 Center for Biomarkers and Biotech Drugs, Kaohsiung Medical University, Kaohsiung 807, Taiwan; 6 Department of Emergency Medicine, Kaohsiung Medical University Hospital, Kaohsiung Medical University, Kaohsiung 807, Taiwan; ijengyeh@hotmail.com (I.-J.Y.); kuantingliu7@gmail.com (K.-T.L.); 7 Graduate Institute of Clinical Medicine, College of Medicine, Kaohsiung Medical University, Kaohsiung 807, Taiwan; 8 School of Medicine, College of Medicine, Kaohsiung Medical University, Kaohsiung 807, Taiwan

**Keywords:** non-coding RNA, transfer RNA (tRNA), RNA sequencing, short-hairpin RNA (shRNA), indoleamine 2,3-dioxygenase1 (IDO1), neutrophil, animal tumor model, biomarker

## Abstract

RNA-based therapeutics are considered as novel treatments for human diseases. Our previous study demonstrated that treatment with short-hairpin RNA against *Ido1* (IDO shRNA) suppresses tumor growth, detects Th1-bias immune responses, and elevates expression of tryptophan transfer RNA (tRNA^Trp^) in total splenocytes. In addition, depletion of Ly6g^+^ neutrophils attenuates the effect of IDO shRNA. The aim of this study was to investigate the regulatory network and the expression profile of tRNAs and other non-coding RNAs in IDO shRNA-treated spleens. The total splenocytes and magnetic bead-enriched splenic neutrophils were collected from the lung tumor bearing mice, which were treated with IDO shRNA or scramble IDO shRNA, and the collected cells were subsequently subjected to RNA sequencing. The gene ontology analysis revealed the different enrichment pathways in total splenocytes and splenic neutrophils. Furthermore, the expression of tRNA genes was identified and validated. Six isoacceptors of tRNA, with different expression patterns between total splenocytes and splenic neutrophils, were observed. In summary, our findings not only revealed novel biological processes in IDO shRNA-treated total splenocytes and splenic neutrophils, but the identified tRNAs and other non-coding RNAs may contribute to developing a novel biomarker gene set for evaluating the clinical efficiency of RNA-based cancer immunotherapies.

## 1. Introduction

RNA-based therapeutics serve as potential treatments for various human diseases. For example, mRNA vaccines, which encode specific antigens, can induce specific immune responses against disease. The effect of multiple mRNA vaccines against infectious diseases and cancers have been evaluated in both animal models and humans [1,2,3]. Silencing disease-related genes has been demonstrated as a strategy to treat various diseases. Antisense oligonucleotide drugs and small interfering RNA (siRNA)-based drugs are approved to treat spinal muscular atrophy, Duchenne muscular dystrophy, and hereditary transthyretin-mediated amyloidosis [4,5]. Moreover, targeting the immune inhibitory genes via siRNA or short-hairpin RNA (shRNA) can induce immune responses against cancers [6,7]. However, the molecular level mechanisms in siRNA/shRNA-targeted immune cells, and the biomarkers for evaluating the therapeutic effect, are not well known.

Indoleamine 2,3-dioxygenase 1 (IDO1) is a tryptophan-degrading enzyme, and an important immune suppressive gene in dendritic cells of tumor-draining lymphoid organs [8,9]. As the kynurenine, which is degraded from tryptophan by IDO1 enzyme activity and downstream derivative metabolites of kynurenine plays, an important role in immune suppression, multiple types of IDO1 inhibitors are developed for cancer treatments [10]. Our previous studies revealed that in vivo delivery of IDO shRNA suppresses tumor growth in several murine tumor models, including the LLC1 lung tumor model [11,12,13]. The effect of IDO shRNA treatment is different from treatment with D-1-methyl-tryptophan (D-1MT), an inhibitor of IDO1. In the LLC1 tumor model, depletion of Ly6g^+^ neutrophils in the spleen abolishes the therapeutic effect of IDO shRNA treatment, but does not affect the therapeutic effect of D-1MT [11]. This may suggest that Ly6g^+^ neutrophils play a critical role in IDO shRNA-mediated antitumor.

Expansion of immature neutrophils can be observed in circulating blood and the spleen of tumor-bearing mice [14]. These immature neutrophils, as well as splenic immature neutrophils, are considered to be immune-suppressive and tumor-supporting neutrophils [15,16,17]. The current evidence reveals that transforming growth factor-β (TGF-β) is a major regulator for immune-suppressive neutrophils, which can be switched to antitumor neutrophils via blockage of TGF-β [18]. However, our previous study revealed that TGF-β may not be a key regulator of splenic neutrophils in IDO shRNA-mediated antitumor responses [11]. Since the tumor-specific cytotoxic T cell can be activated by IDO shRNA in spleen [12,13], the signaling pathways of splenic immune cells are worthy of being further investigated.

Furthermore, the expression of tryptophan tRNA (tRNA^Trp^) was increased in IDO shRNA-treated total splenocytes [11], but the function and regulation of tRNA^Trp^ was unknown. The physiology functions of many non-coding RNAs were identified, and some of these non-coding RNAs can be used as therapeutic targets, and prognostic and diagnostic biomarkers for several types of cancers [19,20]. We wondered whether the expression of tRNAs, and other non-coding RNAs in immune cells, was associated with IDO shRNA treatment and its antitumor effect. This study aimed to investigate the novel signaling pathways, and the expression profile of protein-coding RNAs and non-coding RNAs, in IDO shRNA-treated total splenocytes and splenic neutrophils in lung tumor-bearing mice.

## 2. Results

### 2.1. The Effect of IDO1 shRHA on LLC1 Tumor-Bearing Mice

**Figure 1:**
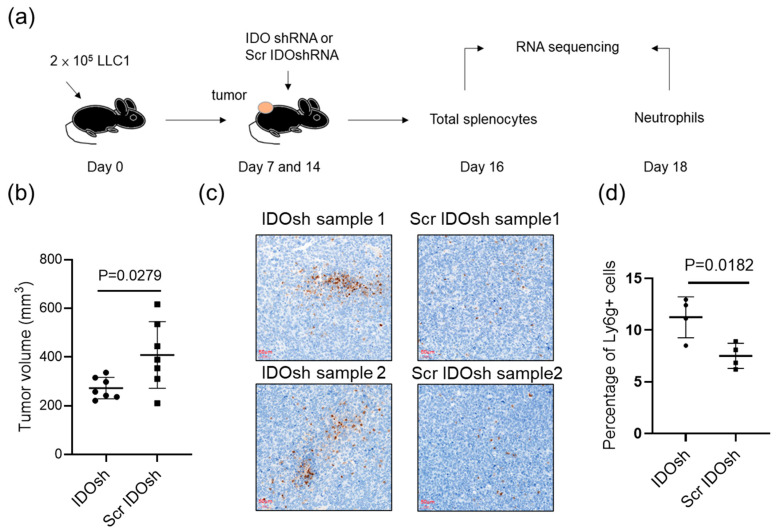
The effect of indoleamine 2,3-dioxygenase (IDO) short-hairpin RNA (shRNA) treatment on LLC1 tumor-bearing mice. (**a**) 2 × 10^5^ LLC1 cells were subcutaneously injected into female inbred C57BL/6JNarl mice. Then, 50 μg of Scramble IDO shRNA (Scr IDOsh) or IDO shRNA (IDO sh) was treated by intramuscular injection at 7 and 14 days after LLC1 injection. The total splenocytes at day 16 and magnetic bead-enriched neutrophils at day 18 were collected, and subsequently subjected to RNA sequencing. (**b**) Mean tumor volume at day 16 (*n* = 7). (**c**) The tumor-infiltrating Ly6g^+^ cells. The labels sample 1 and sample 2 indicate that the images were taken from two independent murine tumors of each group. (**d**) Quantification of Ly6g^+^ cells for immunohistochemistry analysis (*n* = 4).

Our previous studies demonstrated that IDO shRNA treatment delayed tumor growth and resulted in an increasing number of tumor-infiltrating neutrophils [11,12,13]. However, the mechanism for its antitumor effect was not fully understood, especially the role of splenic neutrophils. To investigate the potential regulatory mechanism in splenic neutrophils and total splenocytes, LLC1-tumor-bearing mice were injected with IDO shRNA or scramble IDO shRNA (Scr IDOsh), and then the total splenocytes and magnetic bead-enriched neutrophils were collected, and then subjected to RNA sequencing (Figure 1a). Similar to our previous studies, the mean tumor volume in IDO shRNA treated-groups was smaller than that in Scr IDO1shRNA-treated groups at day 16, post injection of LLC1 tumor cells (Figure 1b). The immunohistochemistry staining analysis confirmed that the IDO shRNA treatment increased the number of Ly6g^+^ tumor-infiltrating neutrophils (Figure 1c,d). The result showed the effect of IDO shRNA treatment in the present study was similar to our previous studies.

### 2.2. The Expression Pattens of Inflammatory Molecules in Total Splencytes and Splenic Neutrophils

**Figure 2:**
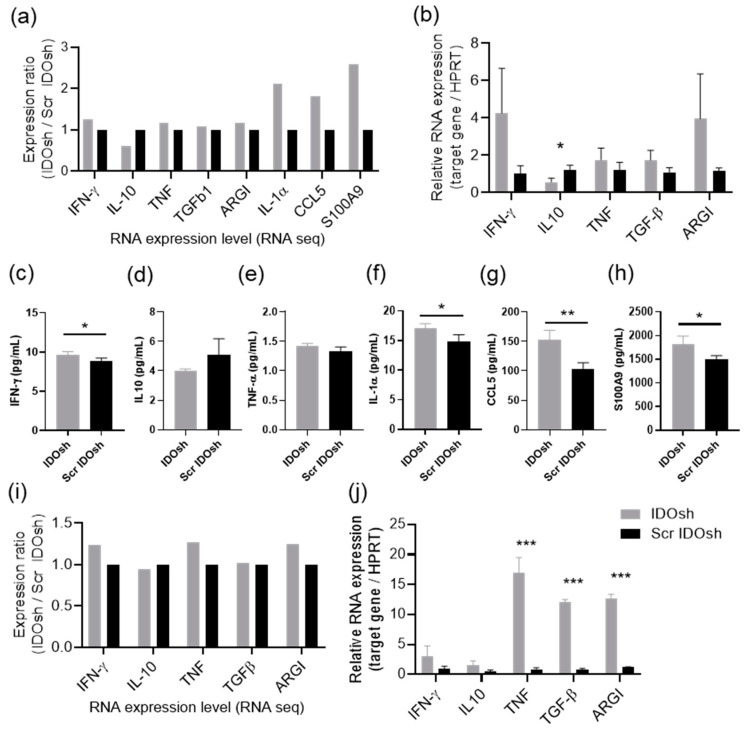
The expression of inflammatory molecules. (**a**) The expression of inflammatory molecules of total splenocytes, determined by RNA sequencing and (**b**) by QPCR. To detect the secreted protein levels, splenocytes were collected at 16 days after LLC1 injection. After 24-h culture, these molecules were detected by magnetic fluorescence microsphere immunoassay (MAGPIX) analysis. The expression of (**c**) IFN-γ, (**d**) IL-10, (**e**) TNF-α, (**f**) IL-1α, (**g**) CCL5, and (**h**) S100A9 was shown. (**i**) The expression of inflammatory molecules of splenic neutrophils determined by RNA sequencing and (**j**) by QPCR. *, **, and *** A statistically significant difference when compared with scramble IDO shRNA group (* *p* < 0.05; ** *p* < 0.01; *** *p* < 0.001). *n* = 3–4 in each experiment, except to RNA sequencing data. IFN-γ, interferon-γ; IL-10, interlukein-10; TNF-α, tumor necrosis factor- α; TGF-β, tumor transforming growth factor-β; CCL5, CC chemokine ligand 5; S100A9, S100 calcium-binding protein A9; Arg1, arginase 1.

Various genes with differential expression were identified via the results of RNA sequencing. The results of extracted RNA quantity assessment and the mapping summary are shown in Supplementary Figure S1. The result of RNA sequencing showed that the expression of IDO in total splenocytes was not significantly affected by IDO shRNA treatment, and was similar to our previous studies [11]. In addition, the expression of several cytokines, including interferon-γ (IFN-γ), tumor necrosis factor-α (TNF-α), interleukin-4 (IL-4), and IL-10, had been determined in the IDO shRNA-treated LLC1-tumor bearing mice in our previous study [11]. To validate the results of RNA sequencing, the inflammatory molecules expression of total splenocytes, determined via RNA sequencing and quantitative PCR (QPCR), are shown in Figure 2a,b, respectively. The protein expression of these inflammatory molecules was further examined in culture medium, which were cultured total splenocytes for 24 h (Figure 2c–h). The present results were comparable to those in our previous study [11]. Moreover, the expression of splenic neutrophils were measured via RNA sequencing and QPCR (Figure 2i,j). The protein expression analysis of splenic neutrophils was not performed because insufficient neutrophils were collected. The expression patterns of most genes identified by RNA sequencing were similar to those of the QPCR analysis in splenic neutrophils.

### 2.3. IDO shRNA-Mediated Pathways in Total Splenocytes and Splenic Neutrophils

**Figure 3:**
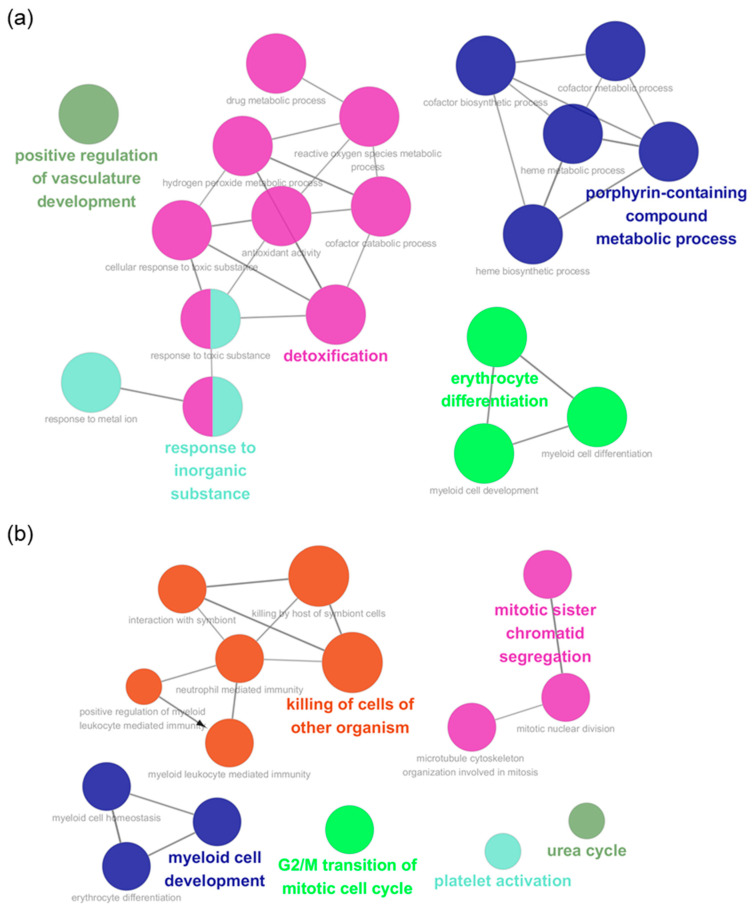
The IDO shRNA-mediated network of biological processes. The Scr IDO shRNA-treated splenocytes or neutrophils, and the identified genes, with differential expression in IDO shRNA-treated cells, were selected, and then the gene ontology analysis was performed via ClueGo and CluePedia. (**a**) The enriched biological processes in IDO shRNA-treated splenocytes (*p* value < 0.001). (**b**) The enriched biological processes in IDO shRNA-treated splenic neutrophils (*p* value < 0.01).

A total of 334 and 237 protein coding genes, with differential expression, were identified from the results of RNA sequencing of total splenocytes and splenic neutrophils, respectively. The full list of these genes is shown in Supplementary Tables S1 and S2. The gene ontology analysis, including immune system process, biological process, molecular function, and cellular component, was performed via ClueGo and CluePedia [21]. The graphical network is shown in Figure 3a,b and the detailed list is shown in Table 1 and Table 2. The results revealed that the significantly enriched biological processes in IDO shRNA-treated total spleen were different from those in IDO shRNA-treated splenic neutrophils. Compared to Scr IDOshRNA-treated total splenocytes, various biological processes, such as porphyrin-containing compound biosynthetic process, detoxification, erythrocyte differentiation, positive regulation of vasculature development, and response to inorganic substance, were enriched in IDO shRNA-treated splenocytes. By contrast, the enriched biological processes, including killing of cells of other organisms, mitotic sister chromatid segregation, myeloid cell development, etc., were observed in IDO shRNA-treated neutrophils.

### 2.4. The Expression Profile of Transfer RNA (tRNA)

**Figure 4:**
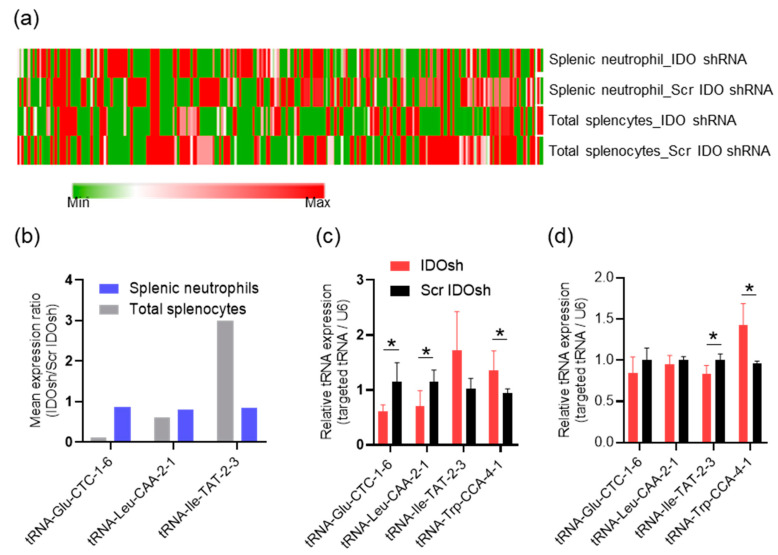
The expression of tRNA genes. (**a**) The heatmap shows the expression of each tRNA gene. Each column indicates a specific tRNA gene. The relative color scheme is based on minimum-maximum values (RPKM), per column. (**b**) The expression of some tRNA genes identified via RNA sequencing in total splenocytes and splenic neutrophils. (**c**) The expression of some tRNA genes identified via QPCR in total splenocytes, and (**d**) in splenic neutrophils. *n* = 3–4 in QPCR analysis. * A statistically significant difference when compared with the Scr IDO shRNA group (* *p* < 0.05).

Apart from the protein coding genes, the transcription of tRNA genes was also determined via RNA sequencing. A total of 237 tRNA genes, which were recognized by codons of 19 types of amino acids and selenocysteine, were identified. In Figure 3a, the heatmap shows the expression patterns of each identified tRNA gene (Figure 4a). However, the tRNA of tryptophan (tRNA-Trp) was not identified via RNA sequencing. To confirm the results of RNA sequencing and determine the expression of tRNA-Trp, the expression of tRNA-GluCTC, tRNA-LeuCAA, tRNA-IleTAT, and tRNA-TrpCCA was recorded, via QPCR. Compared to the results of RNA sequencing (Figure 4b), the QPCR-determined expression of the other tRNA genes was generally similar (Figure 4c,d). Similarly to our previous study, a higher expression of tRNA-Trp was detected in IDO shRNA-treated splenocytes and neutrophils, when compared to Scr IDO shRNA-treated splenocytes and neutrophils. In addition, 56 tRNA genes in total splenocytes, and 67 tRNA in splenic neutrophils genes, with a mean expression ratio of >2 or <0.5, were observed. The 30 identical tRNA genes in both cells are shown in Table 3.

### 2.5. The Potential Regulatory Mechanism of tRNA Transcription

**Figure 5:**
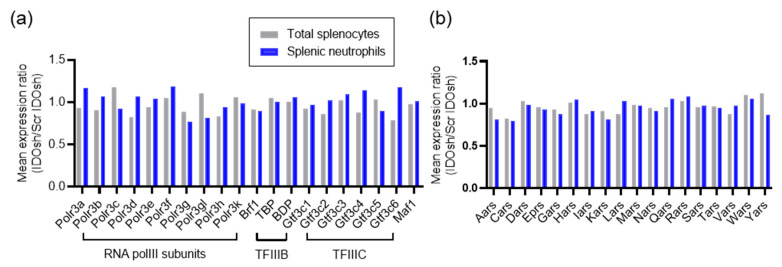
The potential regulatory genes of tRNA transcription. (**a**) The expression of RNA polymerase III machinery, including the subunits of RNA polymerase III (RNA polIII), transcription factor for polymerase III B (TFIIIB), and TFIIIC. (**b**) The expression of aminoacyl-tRNA synthetase genes. The official gene symbol of each aminoacyl-tRNA synthetase gene is shown on the label of *x*-axis in this figure.

The transcription of tRNA was regulated by RNA polymerase III. The results showed that only a part of the tRNA genes expression was significantly changed after IDO shRNA treatment; we supposed that the RNA polymerase III machinery was not altered. The expression of RNA polymerase III machinery, including the subunit of RNA polymerase III, the transcription factor for polymerase III B (TFIIIB), and TFIIIC, was not significantly altered by IDO shRNA treatment in both cells (Figure 5a). Moreover, aminoacyl-tRNA synthetases are a group of enzymes which pair tRNAs with their cognate amino acids [22]. However, IDO shRNA treatment did not affect the transcriptional change of these aminoacyl-tRNA synthetases genes in RNA sequencing data (Figure 5b). Therefore, this may imply that the transcription of these tRNA genes is regulated via other mechanisms, but not common tRNA regulatory mechanisms.

### 2.6. The Fragments Derived from tRNAs

The fragments derived from tRNAs (tRFs) are a type of small non-coding RNA, which derive from tRNA and are considered as biomarkers for human diseases or regulators for biological functions [23,24]. Since the transcription of specific tRNA genes was affected by IDO shRNA treatment, the tRFs derived from these tRNA were supposed to be affected as well. The potential mouse tRFs were evaluated via the tRFdb database [25], and the 5′-tRFs and 3′-tRFs, related to the tRNA identified in Table 3, are shown in Table 4 and Table 5, respectively. As the QPCR data indicated that expression of tRNA-Trp-CCA-4-1 in the IDO shRNA treated group was higher than that in the Scr IDO shRNA treated group, the 3′-tRF of tRNA-Trp-CCA-4-1 was listed.

### 2.7. The Other Types of Non-Coding RNAs

**Figure 6:**
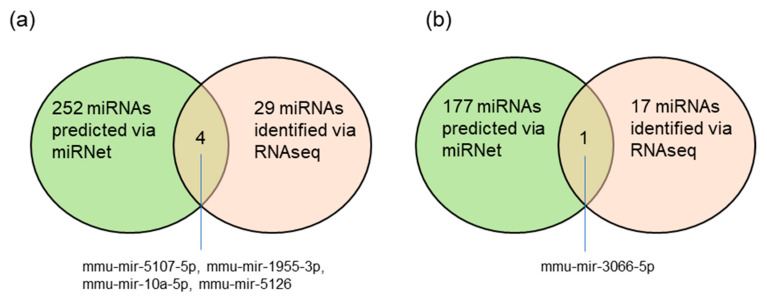
Venn diagrams indicating the shared miRNA genes between miRNet-predicted miRNAs and RNA sequencing-identified miRNAs. (**a**) Total splenocytes. (**b**) Splenic neutrophils.

Except for the tRFs, the other types of non-coding RNAs, such as RIKEN cDNA genes, small nucleolar RNA genes, and microRNA (miRNA) genes were identified by RNA sequencing. The RNA sequencing respectively identified 29 and 17 miRNA genes, in total splenocytes and splenic neutrophils, according to the criteria expression ratio > 2 or <0.5, and RPKM > 0.1. However, the function of these non-coding RNAs in IDO shRNA-affected immune cells was not understood. To investigate the potential interactions between miRNAs and protein coding genes, the protein-coding genes-targeted miRNAs were predicted, via the miRNet website [26]. According to the, 334 and 237 protein coding genes, respectively, identified from total splenocytes and splenic neutrophils, 252 and 177 miRNAs were predicted to target the protein-coding genes with differential gene expression. In Figure 6a,b, the Venn diagrams show that four miRNAs (mmu-mir-5107-5p, mmu-mir-1955-3p, mmu-mir-10a-5p, mmu-mir-5126) and one miRNA (mmu-mir-3066-5p) were observed in both the predicted miRNA list and the result of RNA sequencing. The full list of miRNAs and other non-coding RNA genes are shown in Supplementary Tables S3 and S4.

## 3. Discussion

Our previous study demonstrated that the depletion of Ly6g^+^ neutrophils abolished the antitumor effect of IDO shRNA treatment in a murine lung tumor model [11]. Thus, we were interested in investigating the role of splenic neutrophils during the treatment of IDO shRNA. Since the tumor growth was significantly inhibited by IDO shRNA at day 16 post tumor cells injection, the total splenocytes were collected at day 16. According to our original experiment design, the total splenocytes and the splenic neutrophils should be collected from the same mice. However, the collected cell number was insufficient to perform RNA sequencing. Therefore, the splenic neutrophils were collected from other mice at day 18 post tumor cells injection. Except for the sample for RNA sequencing, the other samples of total splenocytes and splenic neutrophils for other experiments (Figure 2 and Figure 4) were collected from the same mice at day 16. According to the results of QPCR analysis, the RNA expression patterns of splenic neutrophils collected at day 16 (QPCR) were similar to those at day 18 (RNA sequencing) (Figure 2).

The function of neutrophils can be polarized to antitumorigenic phenotype (N1) or protumorigenic phenotype (N2), and influences immune responses in the tumor environment [27]. Therefore, induction of N1 polarization should be beneficial for improving the efficiency of tumor treatments. A recent study has demonstrated that primary human neutrophils, toward N1 and N2 phenotypes, are feasible in vitro [28]. As neutrophils depletion attenuated the antitumor effect of IDO shRNA [11], we hypothesized that IDO shRNA treatment may affect the polarization of neutrophils in spleen. High expression of intercellular adhesion molecule 1 and TNF-α, and low expression of Arg1 are characteristics of N1 antitumorigenic neutrophils [18,29]. However, our results revealed that the splenic neutrophils did not express classic markers of N1 and N2 phenotypes at this time point (Figure 2). Based on the gene ontology analysis, the positive regulation of neutrophils or myeloid leukocytes-mediated immunity, was observed (Table 2). Therefore, we still supposed that the splenic neutrophils play roles in activating antitumor immune responses, through other mechanisms in the IDO shRNA-treated spleen.

The present study did not investigate each type of immune cell in the IDO shRNA-treated spleen, but the transcriptome of total splenocytes. In Table 1, various enriched pathways involved in regulating the porphyrin-related pathways are shown. Tryptophan (Trp) degrading enzymes, IDO and tryptophan 2,3-dioxygenase (TDO), were considered to affect the porphyrin-related pathways [30]; however, the expression of IDO and TDO was not significantly changed in total splenocytes and splenic neutrophils. This may imply the downstream metabolic pathway of tryptophan was still affected after IDO shRNA treatment. In addition, some enriched pathways in total splenocytes were involved in the process of reactive oxygen species (ROS). Recent evidence indicated that ROS are not only a trigger for activating cell death signaling, but an important regulator for controlling T cell signaling and metabolism [31]. Thus, the role of ROS-related pathways in IDO shRNA-mediated antitumor immune responses needs to be further investigated.

Transfer RNAs are products of RNA polymerase III. We expected that the RNA polymerase III machinery should not be affected by IDO shRNA treatment, because the results of RNA sequencing revealed that the expression of tRNA genes was not globally affected (Figure 5a). Previous studies showed that several types of aminoacyl-tRNA synthetases, including tryptophanyl tRNA synthetase (WRS), lysyl-tRNA synthetase (KRS), and glycyl-tRNA synthetase (GRS) were involved in regulation of immune responses [32,33]. For example, secretion of full-length WRS results in induction of antiviral immunity in immune cells [34]. In immune thrombocytopenia, increased WRS expression in CD4^+^ and CD8^+^ T cells may enhance survival of autoreactive T cells [35]. As the elevated expression of tRNA^Trp^ was detected in total splenocytes and splenic neutrophils, we supposed that the WRS expression may be associated with the expression of tRNA^Trp^, and that the expression of other types of aminoacyl-tRNA synthetases may be affected by IDO shRNA treatment. However, the expression of all aminoacyl-tRNA synthetases was not significantly changed (expression ration <2 or >0.5, Figure 5b). Thus, the results suggested that the tRNAs with differential expression were not directly associated with the expression of aminoacyl-tRNA synthetases.

The tRNA-derived fragments, which were cleavage from precursor tRNAs and mature tRNAs, play roles in physiological processes [23,24]. Four types of tRFs, including 5′-tRFs, 3′-tRFs, 1′-tRFs, and 2′-tRFs, have been identified [36]. After stimulation of anti-CD3 and anti-CD28 antibodies, the tRNA fragments in the primary T cell-secreted extracellular vesicles play a role in the inhibition of T cell activation [37]. Moreover, six types of tRNA fragments, including GlyGCC, GluCTC, GluTTC, GluCTG, LysCTT, and LysTTT, can be mapped to the extracellular vesicles-secreted primary mouse dendritic cells. In addition, lipopolysaccharide or 1α,25-dihydroxyvitamin D3 stimulation did not affect the expression of the six tRNA isoacceptors [38]. The present study showed the six isoacceptors of tRNA (GlyTCC, GlyGCC, AspGTC, GluTTC, ValCAC, and LysCTT), with different expression profiles between total splenocytes and splenic neutrophils. The known tRNA fragments of the six tRNA isoacceptors and TrpCAA are listed in Table 4 and Table 5. We will try to evaluate the function of these tRNA fragments in immune cells and secreted extracellular vesicles in the future.

The other types of small non-coding RNAs were also identified. However, the roles of most non-coding RNA in immune responses was not understood. In addition, the potential miRNA-mediated network was not validated by experimental evidence. Recent studies have determined the physiological roles of the small non-coding immune cells [39,40]. However, the funtions of the tRNAs and non-coding RNAs identified in this study were mainly unknown. Due to the antitumor effect of IDO shRNA, we supposed that these non-coding RNAs may be associated with positive regulation of antirumor immunity, and served as biomarkers for evaluating the therapeutic effect of shRNA-based cancer treatment. Our previous studies demonstrated that shRNA against *Ido1*, *Foxo3*, *Thbs1*, and *Clec4a2* can enhance the antitumor effect of Her2/neu DNA vaccine in the murine tumor model [12,41,42,43]. We will try to investigate whether these non-coding RNA can serve as convincing biomarkers for shRNA-mediated therapeutics.

In summary, the present study revealed that IDO shRNA treatment resulted in positive regulation of neutrophil immunity, and ROS-related pathways in total splenocytes. Furthermore, various non-coding RNAs were identified and may be a novel biomarker gene set for evaluating the shRNA-based antitumor treatment via skin administration or intramuscular injection. RNA technology-mediated cancer immunotherapies have been applied in preclinical studies and clinical trials [6,44]. Even though the route of administration for RNA technology-mediated cancer immunotherapies is different from our present study, our finding provides a novel biomarker gene set for these RNA-based cancer therapeutics.

## 4. Materials and Methods

### 4.1. Cell Culture

LLC1 (LL/2) was purchased from the Bioresource Collection and Research Center (Hsinchu, Taiwan). LLC1 was cultured by Dulbecco’s modified Eagle’s medium (Lonza, Walkersville, MD, USA) containing 10% fetal bovine serum and penicillin/streptomycin (100 U/0.1 mg/mL) (Life Technologies, Grand Island, NY, USA).

### 4.2. Plasmid Preparation

IDO shRNA (IDOsh) plasmid (specificly targeting *Ido1*) and Scramble IDO shRNA (Scr IDOsh) were described in our previous study [12]. The DNA plasmid was prepared via Plasmid DNA MaxiPrep Kit (Norgene, St. Catharines, ON, Canada) according to the manufacturer’s instructions. Both DNA plasmids were dissolved in physiological saline at a concentration of 0.5 μg/μL.

### 4.3. Animal Tumor Model and Therapautic Effect

All the animal experiments in this study were approved by the Animal Care and Use Committee at the Kaohsiung Medical University. All mice were held by the Center for Laboratory Animals of Kaohsiung Medical University, which was accredited by Association for Assessment and Accreditation of Laboratory Animal Care International (AAALAC). To establish a subcutaneous tumor, 2 × 10^5^ cells, which were suspended in 200 μL serum-free DMEM, were injected subcutaneously into six- to eight-week old female inbred C57BL/6JNarl mice (National Laboratory Animal Center, Taiwan). The mice were received with intramuscular injection (50 μg of IDO shRNA or scramble IDO shRNA in 100 μL saline) at day 7 and 14 post LLC1 injection. Tumor size was measured by using calipers every 3–4 days, and tumor volume was calculated according to the formula: (shortest diameter)^2^ × (longest diameter) × 0.5236. For RNA sequencing, 4 mice were used in each group. For the following experiments, including immunohistochemical analysis, QPCR analysis, and soluble factors analysis, 7 mice were used in each group. All mice were injected with LLC1 cells and then developed subcutaneous tumors.

### 4.4. Immunohistochemical Analysis of Ly6g^+^ Cells

LLC1 tumor samples were collected at day 16, post tumor injection. Four tumor samples were obtained from IDO shRNA and Scr IDO shRNA-treated tumor-bearing mice, respectively. The formalin-fixed, paraffin-embedded section of tumor lesions were then analyzed using immunohistochemistry. The Ly6g^+^ cells were detected by anti-Ly6g antibody (Taiclone, Taiwan, Cat.: tcea21879, 1:300 dilution). The immunoreactivity was visualized using a diaminobenzidine (DAB) substrate (DAKO, Denmark), subsequently counterstained with hematoxylin. Mouse spleen samples were used for positive control, and Rabbit IgG (Abcam, Cambridge, UK, Cat.: ab172730, diluted to 1.2 μg/mL) were used for negative control. The images were scanned via 3DHISTECH Panoramic Desk II DW. Images were chosen from three random fields by CaseViewer software (3DHISTECH), and then the images were determined by Image J 1.52 version (NIH, Bethesda, MD, USA) with a plugin named “IHC Profiler” [45].

### 4.5. Detection of Soluble Factors

The soluble factors were measured via Magnetic Luminex^®^ Assays (Mouse Premixed Milti-Analyte Kit, R&D Systems, Minneapolis, MN, USA) on a Luminex MagPix^®^ instrument (Luminex). Briefly, 2 × 10^6^ of total spleen cells were cultured in a 24-well plate with 1000 μL RPMI 1640, and supplied with 10% FBS and penicillin/streptomycin for 24 h. After removing cells, the culture medium was collected and stored at −80 °C until performing experiments. The data were calculated using Milliplex Analyst 5.1 Software (Viagene Tech, Carlisle, MA, USA) according to a five-parameter logistic curve fit curve method.

### 4.6. Collection of Total Splenocytes and Enrichment of Neutrophils

The spleen was collected from IDO shRNA- and Scr IDO shRNA-treated mice at day 16 and 18, post injection of LLC1 cells. Total splenocytes were minced with scissors, digested in spleen dissociation medium (Stem Cell Technologies, Vancouver, BC, Canada), and then removed red blood cells via ammonium chloride solution (Stem Cell Technologies, Vancouver, BC, Canada) using the recommended protocols. To further enrich mouse neutrophils from total spleen cells, an EasySep™ Mouse Neutrophil Enrichment Kit (Stem Cell Technologies, Vancouver, BC, Canada) was used according to manufacturer’s instructions. The purity of sorted cells was routinely more than 80%.

### 4.7. RNA Sequencing

The total splenocytes and neutrophils were collected from two IDO shRNA- or Scr IDO shRNA-treated mice. The pooled cells were homogenized in TRIzol reagent (Thermo Fisher Scientific, Inc., Carlsbad, CA, USA) and then total RNA was extracted. RNA sequencing was technically provided by the Center of Genomic Medicine, National Cheng Kung University. In brief, the library preparation was performed with the NEBNext^®^ Ultra™ Directional RNA Library Prep Kit for Illumina (NEW ENGLAND BioLabs, Cat.: #E7420). Subsequently, the qualified libraries were analyzed by FRAGMENT ANALYZER™ Automated CE System (Advanced Analytical Technologies, Inc., Ankeny, IA, USA) and quantified by Qubit Fluorometer (ThermoFisher). The libraries were sequenced with the Illumina sequencing platform. Mapping and whole transcriptome analysis was performed via TopHat [46] and Bowtie2 [47] software package, mapped to the Mouse genome (mm10), respectively. The sequence of mapped tRNA genes was further examined by GtRNAdb [25] and Genomic tRNA database [48].

### 4.8. Gene ontology Analysis

The criteria of differential gene expressions was set at gene expression ratio of >2 or <0.5, and reads per kilobase million (RPKM) >0.1. For performing the gene ontology analysis and drawing the graphical network, Cytoscape software version 3.7.1 was used, with APPs “ClueGO (version 2.5.7)” [21,49]. Briefly, the gene ontology in ClueGo was set as the “functional analysis” mode, visual style “groups”, ontologies/pathways “GO-BiologicalProcess”, “GO-CellularComponent”,” GO-MolecularFunction”, and ”GO-ImmuneSystemProcess”. The pathways with *p* value < 0.01 (splenic neutrophils) and *p* value < 0.001 (total splenocytes) were shown, respectively. The network specificity was set at “Go Tree Interval” from 3 min level to 4 max level.

### 4.9. Quantitative Real-Time Polymerase Chain Reaction (QPCR)

Total RNA was extracted with a Total RNA Purification Kit (Norgene, St. Catharines, ON, Canada). For determining mRNA and tRNA, complementary DNA (cDNA) was performed using a Prime-Script RT Reagent Kit (Clontech, Kusatsu, Shiga, Japan). All real-time PCR were run on a QuantStudio 5 Real-Time PCR system (Applied Biosystems) using the PrimeScript RT reagent kit (TaKaRa BIO, Shiga, Japan) at the program of 95 °C for 20 s, and then 40 cycles of 95 °C for 3 s, and 60 °C for 30 s. The relative mRNA expression was normalized to the expression of internal control hypoxanthine phosphoribosyltransferase (HPRT) by 2^−∆∆Ct (cycle time)^ method [50]. For determining the expression of tRNA genes, the primers were designed according to the tRNA sequences adapted from tRFdb [25]. U6 small nuclear RNA served as the internal control. The calculation of relative tRNA expression was the same as that of relative mRNA expression. A total of 3-4 replicates were used per sample in the QPCR assay. The primer sequences were listed in Table 6.

### 4.10. Statistical Analysis

All of the numerical data and graphs were generated by GraphPad Prism v8.4.3 (GraphPad Software, San Diego, CA, USA). Data represent mean ± SD. Unpaired two-tailed Student’s *t*-test was used for analysis of the difference between two groups, and a *p* value < 0.05 was considered to indicate a statistically significant difference.

## 5. Conclusions

RNA-based therapeutics provide novel and important strategies to treat cancer. The present study performed the transcriptomic analysis for total spleen and splenic neutrophils. Except for the enriched biological pathways, the tRNAs and other non-coding RNAs with significant different expression were identified. This knowledge may be beneficial for understanding regulatory mechanisms of IDO shRNA or other shRNA-based antitumor treatments in immune cells, especially in splenic neutrophils. These data may contribute to developing a novel biomarker gene set for evaluating the clinical efficiency of cancer immunotherapies.

## Figures and Tables

**Table 1:** The gene ontology enrichment analysis of total splenocytes.

Gene Ontology Term	Count	*p* Value ^1^
GO: 0006778~porphyrin-containing compound metabolic process	13	1.77 × 10^−12^
GO: 0006779~porphyrin-containing compound biosynthetic process	11	1.09 × 10^−11^
GO: 0042168~heme metabolic process	10	3.48 × 10^−09^
GO: 0098754~detoxification	17	3.93 × 10^−09^
GO: 0051186~cofactor metabolic process	28	5.38 × 10^−09^
GO: 0048821~erythrocyte development	11	6.56 × 10^−09^
GO: 0046501~protoporphyrinogen IX metabolic process	7	7.39 × 10^−09^
GO: 0010035~response to inorganic substance	34	1.30 × 10^−08^
GO: 0009636~response to toxic substance	33	1.82 × 10^−08^
GO: 0030218~erythrocyte differentiation	16	2.98 × 10^−08^
GO: 0002262~myeloid cell homeostasis	18	4.65 × 10^−08^
GO: 1990748~cellular detoxification	15	5.21 × 10^−08^
GO: 0061515~myeloid cell development	13	7.53 × 10^−08^
GO: 0006783~heme biosynthetic process	8	8.70 × 10^−08^
GO: 0097237~cellular response to toxic substance	21	1.13 × 10^−07^
GO: 0055076~transition metal ion homeostasis	15	2.17 × 10^−07^
GO: 0016209~antioxidant activity	13	3.79 × 10^−07^
GO: 0051188~cofactor biosynthetic process	17	7.02 × 10^−07^
GO: 0004869~cysteine-type endopeptidase inhibitor activity	11	1.97 × 10^−06^
GO: 0048872~homeostasis of number of cells	21	3.61 × 10^−06^
GO: 0010038~response to metal ion	23	8.59 × 10^−06^
GO: 0004857~enzyme inhibitor activity	22	3.90 × 10^−05^
GO: 0017144~drug metabolic process	27	4.02 × 10^−05^
GO: 0004601~peroxidase activity	9	4.48 × 10^−05^
GO: 0051187~cofactor catabolic process	9	5.89 × 10^−05^
GO: 0046394~carboxylic acid biosynthetic process	18	1.12 × 10^−04^
GO: 0015669~gas transport	6	1.17 × 10^−04^
GO: 0034755~iron ion transmembrane transport	6	1.17 × 10^−04^
GO: 0045861~negative regulation of proteolysis	21	1.56 × 10^−04^
GO: 0030099~myeloid cell differentiation	21	2.62 × 10^−04^
GO: 0042743~hydrogen peroxide metabolic process	8	3.37 × 10^−04^
GO: 1904018~positive regulation of vasculature development	14	4.16 × 10^−04^
GO: 0032787~monocarboxylic acid metabolic process	26	4.61 × 10^−04^
GO: 0070541~response to platinum ion	3	4.46 × 10^−04^
GO: 0072593~reactive oxygen species metabolic process	17	5.32 × 10^−04^
GO: 0004866~endopeptidase inhibitor activity	14	9.98 × 10^−04^

^1 ^*p* value was corrected with Bonferroni step down, according to ClueGO. The gene ontology term value < 0.001 is shown in this Table.

**Table 2:** The Gene ontology enrichment analysis of splenic neutrophils.

Gene Ontology Term	Count	*p* Value ^1^
GO: 0031640~killing of cells of other organism	7	2.77 × 10^−04^
GO: 0051873~killing by host of symbiont cells	5	3.41 × 10^−04^
GO: 1904018~positive regulation of vasculature development	14	4.16 × 10^−04^
GO: 0002444~myeloid leukocyte mediated immunity	8	7.59 × 10^−04^
GO: 0000070~mitotic sister chromatid segregation	9	1.26 × 10^−03^
GO: 0061515~myeloid cell development	7	1.33 × 10^−03^
GO: 0000086~G2/M transition of mitotic cell cycle	8	1.44 × 10^−03^
GO: 0002446~neutrophil mediated immunity	5	2.01 × 10^−03^
GO: 0051702~interaction with symbiont	7	2.70 × 10^−03^
GO:0030218~erythrocyte differentiation	8	3.12 × 10^−03^
GO:0008519~ammonium transmembrane transporter activity	3	3.68 × 10^−03^
GO:1902850~microtubule cytoskeleton organization involved in mitosis	8	4.26 × 10^−03^
GO:0002262~myeloid cell homeostasis	9	4.30 × 10^−03^
GO:0140014~mitotic nuclear division	11	4.38 × 10^−03^
GO:0006335~DNA replication-dependent nucleosome assembly	4	5.15 × 10^−03^
GO:0002888~positive regulation of myeloid leukocyte mediated immunity	5	6.90 × 10^−03^
GO:0030168~platelet activation	6	8.14 × 10^−03^
GO:0000050~urea cycle	3	8.24 × 10^−03^

^1 ^*p* value was corrected with Bonferroni step down, according to ClueGO. The gene ontology term value < 0.01 is shown in this Table.

**Table 3:** The tRNA genes with different expression patterns, in both total splenocytes and splenic neutrophils.

GtRNAdb Gene Symbol	Locus	Mean Expression Ratio ^1^	Mean Expression Ratio
		Total Splenocytes	Splenic Neutrophils
tRNA-Gly-TCC-1-4	chr1:171079948-171080019 (−)	5.98 × 10^6^	4.05 × 10^−04^
tRNA-Gly-TCC-1-2	chr1:171064699-171064770 (−)	2.48 × 10^0^	7.58 × 10^−02^
tRNA-Asp-GTC-1-2	chr1:171065358-171065429 (−)	1.21 × 10^−02^	4.05 × 10^−04^
tRNA-Asp-GTC-1-1	chr1:171037029-171037100 (−)	2.23 × 10^−01^	7.54 × 10^−02^
tRNA-Glu-TTC-2-1	chr1:34434812-34434883 (−)	2.75 × 10^−01^	3.58 × 10^−01^
tRNA-Gly-GCC-2-1	chr1:171044985-171045055 (+)	5.94 × 10^−02^	1.55 × 10^11^
tRNA-Gly-GCC-1-1	chr1:171066631-171066701 (+)	1.09 × 10^−07^	1.22 × 10^−06^
tRNA-Val-CAC-2-1	chr1:171111443-171111515 (+)	2.81 × 10^−01^	1.47 × 10^−01^
tRNA-Asp-GTC-1-8	chr10:93452978-93453049 (−)	1.15 × 10^−01^	5.80 × 10^−02^
tRNA-Gly-GCC-2-6	chr11:69118794-69118864 (−)	1.26 × 10^3^	4.69 × 10^−06^
tRNA-Val-CAC-2-3	chr11:48818542-48818614 (+)	1.76 × 10^4^	5.40 × 10^3^
tRNA-Glu-TTC-1-3	chr13:23463535-23463606 (+)	8.99 × 10^5^	6.39 × 10^7^
tRNA-Gly-GCC-2-8	chr13:23517203-23517273 (+)	4.23 × 10^−01^	2.21 × 10^−04^
tRNA-Gly-GCC-2-7	chr13:21710580-21710650 (+)	2.77 × 10^−05^	2.49 × 10^−02^
tRNA-Val-CAC-2-4	chr13:22026548-22026620 (+)	1.76 × 10^4^	1.47 × 10^−01^
tRNA-Asp-GTC-1-12	chr13:21935497-21935568 (−)	5.91 × 10^−02^	3.14 × 10^−02^
tRNA-Glu-TTC-1-4	chr14:76152774-76152845 (+)	8.99 × 10^5^	1.81 × 10^1^
tRNA-Glu-TTC-2-2	chr14:79481668-79481739 (+)	2.50 × 10^−05^	1.12 × 10^2^
tRNA-Lys-CTT-3-5	chr17:23533962-23534034 (+)	2.77 × 10^−01^	1.15 × 10^2^
tRNA-Lys-CTT-3-7	chr17:23547360-23547432 (+)	9.35 × 10^7^	1.15 × 10^2^
tRNA-Gly-GCC-2-2	chr2:57182394-57182464 (−)	2.57 × 10^2^	5.62 × 10^−06^
tRNA-Val-CAC-2-2	chr3:96332740-96332812 (+)	1.76 × 10^4^	1.47 × 10^−01^
tRNA-Lys-CTT-3-1	chr3:96428235-96428307 (+)	1.87 × 10^8^	1.06 × 10^−09^
tRNA-Gly-GCC-2-3	chr3:84229011-84229081 (−)	1.58 × 10^−04^	2.05 × 10^3^
tRNA-Asp-GTC-1-7	chr5:125409016-125409087 (−)	1.24 × 10^−01^	7.85 × 10^−02^
tRNA-Asp-GTC-1-6	chr5:125405607-125405678 (−)	1.24 × 10^−01^	3.60 × 10^1^
tRNA-Val-CAC-4-1	chr6:10100371-10100443 (+)	1.03 × 10^6^	1.71 × 10^−06^
tRNA-Glu-TTC-1-1	chr7:58399315-58399386 (+)	8.99 × 10^5^	1.81 × 10^1^
tRNA-Gly-GCC-2-5	chr8:110631246-110631316 (−)	3.88 × 10^0^	2.22 × 10^8^
tRNA-Gly-GCC-2-4	chr8:110630553-110630623 (−)	1.95 × 10^6^	4.54 × 10^−01^

^1^ Mean expression ratio (IDO shRNA/Scr IDO shRNA) > 2 or <0.5.

**Table 4:** The list of 5′-tRFs.

GtRNAdb Gene Symbol	tRF ID	Type	Sequence
tRNA-Gly-GCC-1-1	5002a 5002b	trf-5	GCATGGGTGGTTCAGTGGTAGA GCATGGGTGGTTCAGTGGTAGAATTCTCGCC
tRNA-Gly-GCC-2-1 tRNA-Gly-GCC-2-6 tRNA-Gly-GCC-2-7 tRNA-Gly-GCC-2-8 tRNA-Gly-GCC-2-2 tRNA-Gly-GCC-2-3	5004a 5004b	trf-5	GCATTGGTGGTTCAGTGGTAGA GCATTGGTGGTTCAGTGGTAGAATTCTCGCC
tRNA-Lys-CTT-3-1 tRNA-Lys-CTT-3-1	5006a 5006b	trf-5	GCCCGGCTAGCTCAGTCGG GCCCGGCTAGCTCAGTCGGTAGAGC
tRNA-Val-CAC-2-1 tRNA-Val-CAC-2-2 tRNA-Val-CAC-2-3 tRNA-Val-CAC-2-4	5019a 5019b	trf-5	GTTTCCGTAGTGTAGTGG GTTTCCGTAGTGTAGTGGTTATCACG
tRNA-Glu-TTC-1-3	5020a 5020b	trf-5	TCCCACATGGTCTAGCGG TCCCACATGGTCTAGCGGTTAGG

**Table 5:** The list of 3′-tRFs.

GtRNAdb Gene Symbol	tRF ID	Type	Sequence
tRNA-Gly-GCC-1-1	3043a 3043b	trf-3	TTCCCGGCCCATGCACCA TCGATTCCCGGCCCATGCACCA
tRNA-Gly-GCC-2-1 tRNA-Gly-GCC-2-2 tRNA-Gly-GCC-2-3 tRNA-Gly-GCC-2-6 tRNA-Gly-GCC-2-7 tRNA-Gly-GCC-2-8	3042a 3042b	trf-3	TTCCCGGCCAATGCACCA TCGATTCCCGGCCAATGCACCA
tRNA-Lys-CTT-3-1 tRNA-Lys-CTT-3-1	3031a 3031b	trf-3	AGCCCCACGTTGGGCGCCA TCGAGCCCCACGTTGGGCGCCA
tRNA-Val-CAC-2-1 tRNA-Val-CAC-2-2 tRNA-Val-CAC-2-3 tRNA-Val-CAC-2-4	3009a 3009b	trf-3	ACCGGGCGGAAACACCA TCGAAACCGGGCGGAAACACCA
tRNA-Glu-TTC-1-3	3029a 3029b	trf-3	CTCCCGGTGTGGGAACCA TCGACTCCCGGTGTGGGAACCA
tRNA-Trp-CCA-4-1	3002a 3002b	trf-3	ATCACGTCGGGGTCACCA TCAAATCACGTCGGGGTCACCA

**Table 6:** Primer sequences.

Target Gene	Primer Sequence
Hypoxanthine phosphoribosyltransferase (HPRT)	Forward: 5′-GTTGGATACAGGCCAGACTTTGTTG-3′ Reverse: 5′-GATTCAACTTGCGCTCATCTTAGGC-3′
IFN-γ	Forward: 5′-AACGCTACACACTGCATCTTGG-3′ Reverse: 5′-CAAGACTTCAAAGAGTCTGAGG-3
TNF-α	Forward: 5′-CCCCAAAGGGATGAGAAGTT-3′ Reverse: 5′-CACTTGGTGGTTTGCTACGA-3′
IL-10	Forward: 5′-CCAGTTTTACCTGGTAGAAGTGATG-3′ Reverse: 5′-TGTCTAGGTCCTGGAGTCCAGCAGACTCAA-3′
TGF-β	Forward: 5′-TGCGCTTGCAGAGATTAAAA-3′ Reverse: 5′-CGTCAAAAGACAGCCACTCA-3
Arginase 1	Forward: 5′-CAGAAGAATGGAAGAGTCAG-3′ Reverse: 5′-CAGATATGCAGGGAGTCACC-3
tRNA-Glu-CTC-1-6	Forward: 5′-TCCCTGGTGGTCTAGTGGTTAG-3′ Reverse: 5′-TTCCCTGACCGGGAATCGAAC-3
tRNA-Leu-CAA-2-1	Forward: 5′-GTCAGGATGGCCGAGTGGTCTAA-3′ Reverse: 5′-TGTCAGAAGTGGGATTCGAACG-3
tRNA-Ile-TAT-2-3	Forward: 5′-GCTCCAGTGGCGCAATCGGTT-3′ Reverse: 5′-TGCTCCAGGTGAGGCTCGAAC-3
tRNA-Trp-CAA-4-1	Forward: 5′-GACCTCGTGGCGCAACGGTA-3′ Reverse: 5′-TGACCCCGACGTGATTTG-3′
U6	Forward: 5′-CGATACAGAGAAGATTAGCATGGC-3′ Reverse: 5′-AACGCTTCACGAATTTGCGT-3′

## Supplementary Materials

Supplementary Materials can be found at https://www.mdpi.com/1422-0067/21/18/6703/s1. Figure S1: Read Quality Distribution with mean quality (in Phred Score) in *x*-axis and number of reads in *y*-axis for samples individually (a) Total splenocytes IDOsh day16. (b) Total splenocytes Scr IDOsh day16. (c) Splenic neutrophils IDOsh day18. (d) Splenic neutrophils Scr IDOsh day18. Phred Score meaning: Quality scores range from 4 to about 60, with higher values corresponding to higher quality. The quality scores are logarithmically linked to error probabilities. (e) The mapping summary of this study; Table S1. The list of protein-coding genes in total splenocytes with significant changes; Table S2. The list of protein-coding genes in splenic neutrophils with significant changes; Table S3. The list of non-coding RNA genes in total splenocytes with significant changes; Table S4. The list of non-coding RNA genes in splenic neutrophils with significant changes.
