# Supplementary material for: The Expression Profile of mRNA and tRNA Genes in Splenocytes and Neutrophils after In Vivo Delivery of Antitumor Short Hairpin RNA of Indoleamine 2,3- Dioxygenase
Source: Int J Mol Sci. 2020 Sep 13;21(18):6703. doi: 10.3390/ijms21186703 (PMC7555719; doi:10.3390/ijms21186703)
Supplement: Supplementary file 1 [file ijms-21-06703-s001.zip › ijms-898817-supplementary.docx]

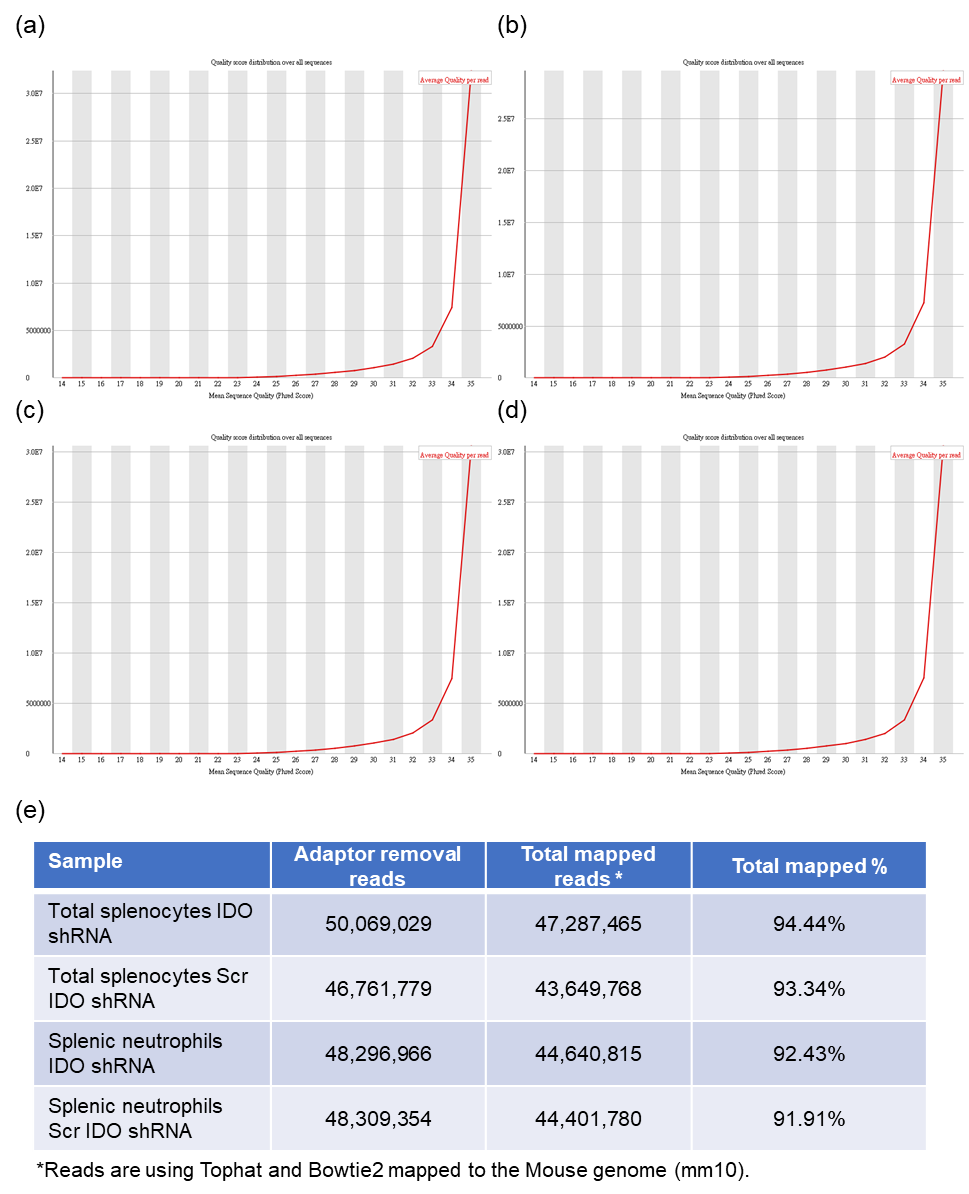


**Supplementary Figure S1.** Read Quality Distribution with mean quality (in Phred Score) in x-axis and number of reads in y-axis for samples individually (**a**) Total splenocytes IDOsh day16. (**b**) Total splenocytes Scr IDOsh day16. (**c**) Splenic neutrophils IDOsh day18. (**d**) Splenic neutrophils Scr IDOsh day18. Phred Score meaning: Quality scores range from 4 to about 60, with higher values corresponding to higher quality. The quality scores are logarithmically linked to error probabilities. (**e**) The mapping summary of this study.

**Supplementary Table S1.** The list of protein-coding genes in total splenocytes with significant changes. (expression ration IDO shRNA / Scr IDO shRNA >2 or < 0.5, RPKM > 0.1)

| **Official gene symbol** | **Description** | **Expression ratio** |
| --- | --- | --- |
| AA467197 | expressed sequence AA467197 | 3.0718 |
| Abcb10 | ATP-binding cassette, sub-family B (MDR/TAP), member 10 | 3.3112 |
| Abcb4 | ATP-binding cassette, sub-family B (MDR/TAP), member 4 | 6.5384 |
| Abcb6 | ATP-binding cassette, sub-family B (MDR/TAP), member 6 | 2.6206 |
| Abcg2 | ATP binding cassette subfamily G member 2 (Junior blood group) | 4.9668 |
| Abhd5 | abhydrolase domain containing 5 | 2.1234 |
| Ache | acetylcholinesterase | 6.9538 |
| Acsl1 | acyl-CoA synthetase long-chain family member 1 | 2.3713 |
| Actl10 | actin-like 10 | 2.0186 |
| Actl7b | actin-like 7b | 0.4681 |
| Adgrg1 | adhesion G protein-coupled receptor G1 | 3.4047 |
| Adgrg3 | adhesion G protein-coupled receptor G3 | 3.4074 |
| Agr2 | anterior gradient 2 | 0.3657 |
| Aif1 | allograft inflammatory factor 1 | 0.4961 |
| Alad | aminolevulinate, delta-, dehydratase | 2.1919 |
| Alas2 | aminolevulinic acid synthase 2, erythroid | 5.8354 |
| Aldh1a1 | aldehyde dehydrogenase family 1, subfamily A1 | 8.8030 |
| Alox12 | arachidonate 12-lipoxygenase | 0.3840 |
| Ank1 | ankyrin 1, erythroid | 28.7903 |
| Ankle1 | ankyrin repeat and LEM domain containing 1 | 2.4396 |
| Anxa1 | annexin A1 | 2.4128 |
| Anxa3 | annexin A3 | 3.0170 |
| Apol11b | apolipoprotein L 11b | 69.168 |
| Aqp1 | aquaporin 1 | 37.0447 |
| Aqp9 | aquaporin 9 | 3.9982 |
| Arhgap23 | Rho GTPase activating protein 23 | 5.1464 |
| Arhgef10l | Rho guanine nucleotide exchange factor (GEF) 10-like | 0.4619 |
| Arhgef39 | Rho guanine nucleotide exchange factor (GEF) 39 | 2.2255 |
| Art4 | ADP-ribosyltransferase 4 | 4.0724 |
| Asb1 | ankyrin repeat and SOCS box-containing 1 | 2.0622 |
| Asmt | acetylserotonin O-methyltransferase | 0.3072 |
| Asns | asparagine synthetase | 4.3335 |
| Ass1 | argininosuccinate synthetase 1 | 0.2342 |
| Atp1b2 | ATPase, Na+/K+ transporting, beta 2 polypeptide | 8.0087 |
| Atp2b4 | ATPase, Ca++ transporting, plasma membrane 4 | 4.4996 |
| Atpif1 | ATPase inhibitory factor 1 | 2.0972 |
| BC100530 | cDNA sequence BC100530 | 0.1116 |
| Bcat1 | branched chain aminotransferase 1, cytosolic | 0.4822 |
| Bcl2l1 | BCL2-like 1 | 3.3901 |
| Birc5 | baculoviral IAP repeat-containing 5 | 2.0045 |
| Blvrb | biliverdin reductase B (flavin reductase (NADPH)) | 2.4540 |
| Bpgm | 2,3-bisphosphoglycerate mutase | 3.6090 |
| C3 | complement component 3 | 2.4804 |
| Calr3 | calreticulin 3 | 0.4937 |
| Camp | cathelicidin antimicrobial peptide | 117.482 |
| Car1 | carbonic anhydrase 1 | 6.3157 |
| Car2 | carbonic anhydrase 2 | 14.1856 |
| Ccdc116 | coiled-coil domain containing 116 | 0.4385 |
| Ccdc18 | coiled-coil domain containing 18 | 2.0061 |
| Ccer2 | coiled-coil glutamate-rich protein 2 | 0.4152 |
| Cd14 | CD14 antigen | 0.4179 |
| Cd177 | CD177 antigen | 5.8543 |
| Cd209a | CD209a antigen | 2.1315 |
| Cd300c2 | CD300C molecule 2 | 0.4641 |
| Cdr2 | cerebellar degeneration-related 2 | 3.8724 |
| Cebpd | CCAAT/enhancer binding protein (C/EBP), delta | 2.0948 |
| Cela1 | chymotrypsin-like elastase family, member 1 | 3.6862 |
| Cep290 | centrosomal protein 290 | 2.0417 |
| Cercam | cerebral endothelial cell adhesion molecule | 2.9987 |
| Ces2g | carboxylesterase 2G | 11.0196 |
| Chdh | choline dehydrogenase | 2.1356 |
| Chil1 | chitinase-like 1 | 2.6604 |
| Chil3 | chitinase-like 3 | 5.3385 |
| Cited4 | Cbp/p300-interacting transactivator, with Glu/Asp-rich carboxy-terminal domain, 4 | 4.7497 |
| Cldn13 | claudin 13 | 41.0747 |
| Clec2f | C-type lectin domain family 2, member f | 0.4388 |
| Clec4a2 | C-type lectin domain family 4, member a2 | 2.6606 |
| Clec9a | C-type lectin domain family 9, member a | 2.2380 |
| Clu | clusterin | 0.37409 |
| Cma1 | chymase 1, mast cell | 2.7584 |
| Cnih2 | cornichon family AMPA receptor auxiliary protein 2 | 0.4198 |
| Cox6b2 | cytochrome c oxidase subunit 6B2 | 6.3192 |
| Cox7a1 | cytochrome c oxidase subunit 7A1 | 0.2926 |
| Cpa3 | carboxypeptidase A3, mast cell | 2.3697 |
| Cpne2 | copine II | 3.5107 |
| Cpox | coproporphyrinogen oxidase | 3.5965 |
| Creg1 | cellular repressor of E1A-stimulated genes 1 | 2.3732 |
| Crip2 | cysteine rich protein 2 | 3.6954 |
| Ctse | cathepsin E | 6.2549 |
| Ctsg | cathepsin G | 6.8750 |
| Cxcl16 | chemokine (C-X-C motif) ligand 16 | 0.4962 |
| Cxxc4 | CXXC finger 4 | 0.1567 |
| Cystm1 | cysteine-rich transmembrane module containing 1 | 0.4787 |
| Daam1 | dishevelled associated activator of morphogenesis 1 | 2.4814 |
| Dand5 | DAN domain family member 5, BMP antagonist | 2.4980 |
| Dennd2c | DENN/MADD domain containing 2C | 2.7082 |
| Dhrs11 | dehydrogenase/reductase (SDR family) member 11 | 4.1579 |
| Dnph1 | 2'-deoxynucleoside 5'-phosphate N-hydrolase 1 | 2.1942 |
| Dpep2 | dipeptidase 2 | 0.4787 |
| Dusp8 | dual specificity phosphatase 8 | 2.3138 |
| Dynlt1b | dynein light chain Tctex-type 1B | 3.2433 |
| Ecm2 | extracellular matrix protein 2, female organ and adipocyte specific | 2.0346 |
| Elane | elastase, neutrophil expressed | 25.703 |
| Eng | endoglin | 2.3577 |
| Eno1 | enolase 1, alpha non-neuron | 2.1033 |
| Epb41 | erythrocyte membrane protein band 4.1 | 2.0713 |
| Epb42 | erythrocyte membrane protein band 4.2 | 22.4331 |
| Epor | erythropoietin receptor | 9.0692 |
| Eps8l1 | EPS8-like 1 | 0.4078 |
| Ermap | erythroblast membrane-associated protein | 18.4456 |
| F11r | F11 receptor | 0.3098 |
| Fads2 | fatty acid desaturase 2 | 2.4265 |
| Fads3 | fatty acid desaturase 3 | 2.3749 |
| Fahd1 | fumarylacetoacetate hydrolase domain containing 1 | 2.1315 |
| Fam220a | family with sequence similarity 220, member A | 2.1387 |
| Fam24a | family with sequence similarity 24, member A | 0.3511 |
| Fam71e1 | family with sequence similarity 71, member E1 | 0.4388 |
| Fbxo30 | F-box protein 30 | 2.0879 |
| Fech | ferrochelatase | 5.4339 |
| Fgfrl1 | fibroblast growth factor receptor-like 1 | 2.0479 |
| Fhit | fragile histidine triad gene | 2.8838 |
| Fn3krp | fructosamine 3 kinase related protein | 2.5239 |
| Fnip2 | folliculin interacting protein 2 | 3.1418 |
| Fsd2 | fibronectin type III and SPRY domain containing 2 | 2.1751 |
| Fzd6 | frizzled class receptor 6 | 0.4608 |
| Gata1 | GATA binding protein 1 | 10.8733 |
| Gatm | glycine amidinotransferase (L-arginine:glycine amidinotransferase) | 5.1274 |
| Gbx1 | gastrulation brain homeobox 1 | 0.2671 |
| Gca | grancalcin | 2.4403 |
| Gclm | glutamate-cysteine ligase, modifier subunit | 2.9181 |
| Gfi1b | growth factor independent 1B | 8.5572 |
| Ggt1 | gamma-glutamyltransferase 1 | 0.3319 |
| Gh | growth hormone | 3.3156 |
| Glrx5 | glutaredoxin 5 | 2.1722 |
| Gm11110 | predicted gene 11110 | 2.2234 |
| Gm13546 | predicted gene 13546 | 2.0479 |
| Gm15133 | predicted gene 15133 | 0.4961 |
| Gm16793 | predicted gene, 16793 | 3.9449 |
| Gm20337 | predicted gene, 20337 | 2.6330 |
| Gm20556 | predicted gene, 20556 | 0.3291 |
| Gm20594 | predicted gene, 20594 | 0.4388 |
| Gm20604 | predicted gene 20604 | 0.3366 |
| Gm20743 | predicted gene, 20743 | 2.2569 |
| Gm31763 | predicted gene, 31763 | 2.7584 |
| Gm41410 | predicted gene, 41410 | 3.3618 |
| Gm5099 | predicted gene 5099 | 0.3614 |
| Gm5483 | predicted gene 5483 | 0.1218 |
| Gm6578 | mitochondrial ribosomal protein L32 pseudogene | 2.1064 |
| Gm9733 | predicted gene 9733 | 0.4388 |
| Gm9895 | predicted gene 9895 | 6.3631 |
| Gm9949 | predicted gene 9949 | 2.3404 |
| Gml2 | glycosylphosphatidylinositol anchored molecule like 2 | 0.4651 |
| Gpbar1 | G protein-coupled bile acid receptor 1 | 0.4286 |
| Gpsm2 | G-protein signalling modulator 2 (AGS3-like, C. elegans) | 3.8209 |
| Gstm5 | glutathione S-transferase, mu 5 | 6.3631 |
| Gstt1 | glutathione S-transferase, theta 1 | 2.1315 |
| Gypa | glycophorin A | 66.5144 |
| H2-L | histocompatibility 2, D region locus L | 0.4939 |
| H2al1m | H2A histone family member L1M | 0.3616 |
| Hagh | hydroxyacyl glutathione hydrolase | 3.0353 |
| Hbb-b1 | hemoglobin, beta adult major chain | 5.0471 |
| Hbb-b2 | hemoglobin, beta adult minor chain | 6.3436 |
| Hbb-bs | hemoglobin, beta adult s chain | 4.9831 |
| Hbb-bt | hemoglobin, beta adult t chain | 6.3436 |
| Hbq1a | hemoglobin, theta 1A | 2.3746 |
| Hcar2 | hydroxycarboxylic acid receptor 2 | 2.0580 |
| Hebp1 | heme binding protein 1 | 4.1250 |
| Hemgn | hemogen | 44.5415 |
| Hist1h4m | histone cluster 1, H4m | 0.3539 |
| Hk3 | hexokinase 3 | 2.1315 |
| Hmbs | hydroxymethylbilane synthase | 6.4127 |
| Hspb9 | heat shock protein, alpha-crystallin-related, B9 | 0.4388 |
| Hus1b | HUS1 checkpoint clamp component B | 3.2474 |
| Icam4 | intercellular adhesion molecule 4, Landsteiner-Wiener blood group | 6.1437 |
| Ifitm1 | interferon induced transmembrane protein 1 | 0.1635 |
| Ifitm5 | interferon induced transmembrane protein 5 | 0.3192 |
| Il13ra1 | interleukin 13 receptor, alpha 1 | 0.4285 |
| Il1a | interleukin 1 alpha | 2.1079 |
| Isg20 | interferon-stimulated protein | 2.8524 |
| Itsn1 | intersectin 1 (SH3 domain protein 1A) | 2.0732 |
| Jdp2 | Jun dimerization protein 2 | 2.0061 |
| Kbtbd6 | kelch repeat and BTB (POZ) domain containing 6 | 2.1754 |
| Kit | KIT proto-oncogene receptor tyrosine kinase | 2.2860 |
| Klf1 | Kruppel-like factor 1 (erythroid) | 12.3709 |
| Klhdc8b | kelch domain containing 8B | 2.1684 |
| Klhl35 | kelch-like 35 | 2.0186 |
| Klra6 | killer cell lectin-like receptor, subfamily A, member 6 | 2.7591 |
| Klrb1b | killer cell lectin-like receptor subfamily B member 1B | 2.2734 |
| Klre1 | killer cell lectin-like receptor family E member 1 | 2.1129 |
| LOC100038947 | signal-regulatory protein beta 1-like | 0.4848 |
| Lcn2 | lipocalin 2 | 4.8989 |
| Ldhd | lactate dehydrogenase D | 0.3713 |
| Lix1l | Lix1-like | 0.4681 |
| Lockd | lncRNA downstream of Cdkn1b | 2.9428 |
| Lsmem1 | leucine-rich single-pass membrane protein 1 | 0.3695 |
| Ltf | lactotransferrin | 45.805 |
| Ly6g | lymphocyte antigen 6 complex, locus G | 2.7153 |
| Lyz1 | lysozyme 1 | 2.5905 |
| Lyz2 | lysozyme 2 | 2.1474 |
| M1ap | meiosis 1 associated protein | 2.0688 |
| Megf9 | multiple EGF-like-domains 9 | 2.1237 |
| Mfsd2b | major facilitator superfamily domain containing 2B | 4.1630 |
| Mgll | monoglyceride lipase | 3.9801 |
| Mgst3 | microsomal glutathione S-transferase 3 | 6.7141 |
| Mki67 | antigen identified by monoclonal antibody Ki 67 | 2.0953 |
| Mmp25 | matrix metallopeptidase 25 | 2.0704 |
| Morn3 | MORN repeat containing 3 | 0.4726 |
| Mpo | myeloperoxidase | 10.0997 |
| Mrgpra2b | MAS-related GPR, member A2B | 2.5453 |
| Mroh6 | maestro heat-like repeat family member 6 | 0.4773 |
| Mrpl23 | mitochondrial ribosomal protein L23 | 0.4070 |
| Ms4a3 | membrane-spanning 4-domains, subfamily A, member 3 | 4.0958 |
| Mt1 | metallothionein 1 | 2.8524 |
| Mt2 | metallothionein 2 | 3.8617 |
| Myh10 | myosin, heavy polypeptide 10, non-muscle | 2.6673 |
| Myl6b | myosin, light polypeptide 6B | 2.0186 |
| Myl9 | myosin, light polypeptide 9, regulatory | 0.4198 |
| Myo1d | myosin ID | 3.1629 |
| Neil3 | nei like 3 (E. coli) | 2.1628 |
| Nfe2 | nuclear factor, erythroid derived 2 | 2.9683 |
| Ngp | neutrophilic granule protein | 32.28 |
| Nhsl2 | NHS-like 2 | 2.2230 |
| Nkx6-2 | NK6 homeobox 2 | 2.3600 |
| Noxa1 | NADPH oxidase activator 1 | 0.4213 |
| Nqo1 | NAD(P)H dehydrogenase, quinone 1 | 5.5585 |
| Nr1h3 | nuclear receptor subfamily 1, group H, member 3 | 0.3677 |
| Nrn1l | neuritin 1-like | 2.8524 |
| Nt5dc2 | 5'-nucleotidase domain containing 2 | 2.2681 |
| Nxpe2 | neurexophilin and PC-esterase domain family, member 2 | 2.4754 |
| Nyx | nyctalopin | 0.4082 |
| Oaz1-ps | ornithine decarboxylase antizyme 1, pseudogene | 0.2344 |
| Oip5 | Opa interacting protein 5 | 2.0414 |
| Orm3 | orosomucoid 3 | 0.4597 |
| Pabpc1l | poly(A) binding protein, cytoplasmic 1-like | 0.4787 |
| Paqr9 | progestin and adipoQ receptor family member IX | 11.2999 |
| Parvb | parvin, beta | 2.3782 |
| Pcbd1 | pterin 4 alpha carbinolamine dehydratase/dimerization cofactor of hepatocyte nuclear factor 1 alpha (TCF1) 1 | 0.4388 |
| Pcyt1b | phosphate cytidylyltransferase 1, choline, beta isoform | 2.2819 |
| Pdia2 | protein disulfide isomerase associated 2 | 2.1649 |
| Pip5k1b | phosphatidylinositol-4-phosphate 5-kinase, type 1 beta | 2.0400 |
| Pklr | pyruvate kinase liver and red blood cell | 13.3113 |
| Plek2 | pleckstrin 2 | 2.7584 |
| Pnp2 | purine-nucleoside phosphorylase 2 | 3.9202 |
| Poln | DNA polymerase N | 0.4968 |
| Prdx2 | peroxiredoxin 2 | 3.0124 |
| Prg2 | proteoglycan 2, bone marrow | 6.4728 |
| Prkar2b | protein kinase, cAMP dependent regulatory, type II beta | 3.2209 |
| Prss27 | protease, serine 27 | 0.4096 |
| Prtn3 | proteinase 3 | 5.0961 |
| Ptdss2 | phosphatidylserine synthase 2 | 3.6778 |
| Rab20 | RAB20, member RAS oncogene family | 0.4528 |
| Rab3il1 | RAB3A interacting protein (rabin3)-like 1 | 8.1915 |
| Rab44 | RAB44, member RAS oncogene family | 3.2730 |
| Rag1 | recombination activating gene 1 | 4.5690 |
| Rangrf | RAN guanine nucleotide release factor | 2.7427 |
| Reln | reelin | 2.8414 |
| Rfx2 | regulatory factor X, 2 (influences HLA class II expression) | 3.8032 |
| Rgs12 | regulator of G-protein signaling 12 | 2.6692 |
| Rhd | Rh blood group, D antigen | 42.0401 |
| Rln3 | relaxin 3 | 2.1942 |
| Rsad2 | radical S-adenosyl methionine domain containing 2 | 5.5126 |
| Rwdd2a | RWD domain containing 2A | 2.2429 |
| S100a1 | S100 calcium binding protein A1 | 2.3697 |
| S100a8 | S100 calcium binding protein A8 (calgranulin A) | 2.2279 |
| S100a9 | S100 calcium binding protein A9 (calgranulin B) | 2.5940 |
| Samd11 | sterile alpha motif domain containing 11 | 3.1509 |
| Samd14 | sterile alpha motif domain containing 14 | 5.9749 |
| Scrn3 | secernin 3 | 2.3204 |
| Selenbp1 | selenium binding protein 1 | 2.8524 |
| Sgms2 | sphingomyelin synthase 2 | 2.0764 |
| Siglech | sialic acid binding Ig-like lectin H | 2.5599 |
| Sirpb1b | signal-regulatory protein beta 1B | 0.4076 |
| Slc15a2 | solute carrier family 15 (H+/peptide transporter), member 2 | 0.1779 |
| Slc16a10 | solute carrier family 16 (monocarboxylic acid transporters), member 10 | 3.6251 |
| Slc22a23 | solute carrier family 22, member 23 | 5.1231 |
| Slc25a37 | solute carrier family 25, member 37 | 3.1250 |
| Slc39a8 | solute carrier family 39 (metal ion transporter), member 8 | 2.1018 |
| Slc43a1 | solute carrier family 43, member 1 | 4.4261 |
| Slc4a1 | solute carrier family 4 (anion exchanger), member 1 | 75.6487 |
| Smim1 | small integral membrane protein 1 | 3.3931 |
| Smox | spermine oxidase | 2.5039 |
| Snca | synuclein, alpha | 4.1851 |
| Snx22 | sorting nexin 22 | 2.6753 |
| Sord | sorbitol dehydrogenase | 2.0897 |
| Specc1 | sperm antigen with calponin homology and coiled-coil domains 1 | 7.7754 |
| Sphk1 | sphingosine kinase 1 | 5.9681 |
| Spire1 | spire type actin nucleation factor 1 | 7.4881 |
| Sptb | spectrin beta, erythrocytic | 20.5288 |
| Ssx2ip | synovial sarcoma, X 2 interacting protein | 3.0881 |
| St3gal5 | ST3 beta-galactoside alpha-2,3-sialyltransferase 5 | 6.4156 |
| St5 | suppression of tumorigenicity 5 | 9.0274 |
| Steap3 | STEAP family member 3 | 3.8682 |
| Steap4 | STEAP family member 4 | 0.2533 |
| Stfa1 | stefin A1 | 0.3236 |
| Stfa2l1 | stefin A2 like 1 | 0.2370 |
| Stfa3 | stefin A3 | 0.2025 |
| Stom | stomatin | 3.8571 |
| Stx2 | syntaxin 2 | 2.8416 |
| Svip | small VCP/p97-interacting protein | 4.2238 |
| Tal1 | T cell acute lymphocytic leukemia 1 | 20.3278 |
| Tas2r108 | taste receptor, type 2, member 108 | 2.4136 |
| Tbxas1 | thromboxane A synthase 1, platelet | 2.3039 |
| Tctex1d4 | Tctex1 domain containing 4 | 0.4764 |
| Tfdp2 | transcription factor Dp 2 | 2.3182 |
| Tfr2 | transferrin receptor 2 | 6.3431 |
| Tfrc | transferrin receptor | 2.1717 |
| Tgfbi | transforming growth factor, beta induced | 0.4318 |
| Tgm1 | transglutaminase 1, K polypeptide | 0.4770 |
| Tmcc2 | transmembrane and coiled-coil domains 2 | 11.8651 |
| Tmem14a | transmembrane protein 14A | 0.4388 |
| Tmem191c | transmembrane protein 191C | 0.3934 |
| Tmem221 | transmembrane protein 221 | 2.3404 |
| Tmod1 | tropomodulin 1 | 8.4139 |
| Tmtc3 | transmembrane and tetratricopeptide repeat containing 3 | 2.2124 |
| Tnfaip2 | tumor necrosis factor, alpha-induced protein 2 | 2.6384 |
| Tnfrsf17 | tumor necrosis factor receptor superfamily, member 17 | 2.8838 |
| Tns1 | tensin 1 | 2.1857 |
| Tom1l1 | target of myb1-like 1 (chicken) | 5.1232 |
| Trcg1 | taste receptor cell gene 1 | 0.2678 |
| Trim10 | tripartite motif-containing 10 | 50.1066 |
| Trim46 | tripartite motif-containing 46 | 2.1393 |
| Trpc2 | transient receptor potential cation channel, subfamily C, member 2 | 0.2556 |
| Tspan33 | tetraspanin 33 | 35.1863 |
| Tst | thiosulfate sulfurtransferase, mitochondrial | 2.2429 |
| Ttc39a | tetratricopeptide repeat domain 39A | 2.0688 |
| Tubb1 | tubulin, beta 1 class VI | 0.4005 |
| Txnrd2 | thioredoxin reductase 2 | 2.0139 |
| Ubac1 | ubiquitin associated domain containing 1 | 2.3324 |
| Ube2c | ubiquitin-conjugating enzyme E2C | 2.1027 |
| Ube2l6 | ubiquitin-conjugating enzyme E2L 6 | 4.5299 |
| Ube2o | ubiquitin-conjugating enzyme E2O | 2.3573 |
| Ugt1a7c | UDP glucuronosyltransferase 1 family, polypeptide A7C | 0.3804 |
| Urod | uroporphyrinogen decarboxylase | 2.3551 |
| Uros | uroporphyrinogen III synthase | 2.3016 |
| Vamp5 | vesicle-associated membrane protein 5 | 2.1722 |
| Vopp1 | vesicular, overexpressed in cancer, prosurvival protein 1 | 2.0462 |
| Wfdc17 | WAP four-disulfide core domain 17 | 0.1986 |
| Wfdc21 | WAP four-disulfide core domain 21 | 2.4642 |
| Wipi1 | WD repeat domain, phosphoinositide interacting 1 | 5.4610 |
| Xkr5 | X-linked Kx blood group related 5 | 2.0186 |
| Xpo7 | exportin 7 | 2.6203 |
| Zcchc3 | zinc finger, CCHC domain containing 3 | 2.6588 |
| Zfp428 | zinc finger protein 428 | 0.4130 |
| Zfyve21 | zinc finger, FYVE domain containing 21 | 0.4486 |

**Supplementary Table S2.** The list of protein-coding genes in splenic neutrophils with significant changes. (expression ration IDO shRNA / Scr IDO shRNA >2 or < 0.5, RPKM > 0.1).

| **Official gene symbol** | **Description** | **Expression ratio** |
| --- | --- | --- |
| AA467197 | expressed sequence AA467197 | 2.0419 |
| Abcg2 | ATP binding cassette subfamily G member 2 (Junior blood group) | 0.4873 |
| Abcg4 | ATP binding cassette subfamily G member 4 | 0.2687 |
| Abhd14a | abhydrolase domain containing 14A | 0.4873 |
| Abhd14b | abhydrolase domain containing 14b | 0.4233 |
| Acat3 | acetyl-Coenzyme A acetyltransferase 3 | 0.4976 |
| Ache | acetylcholinesterase | 0.3603 |
| Acsm4 | acyl-CoA synthetase medium-chain family member 4 | 0.4641 |
| Add2 | adducin 2 (beta) | 0.3836 |
| Adgrg1 | adhesion G protein-coupled receptor G1 | 0.4802 |
| Agbl1 | ATP/GTP binding protein-like 1 | 2.6162 |
| Alad | aminolevulinate, delta-, dehydratase | 0.4524 |
| Alox15 | arachidonate 15-lipoxygenase | 2.1751 |
| Angpt1 | angiopoietin 1 | 0.4811 |
| Ankle1 | ankyrin repeat and LEM domain containing 1 | 0.3329 |
| Anxa3 | annexin A3 | 3.8286 |
| Aqp1 | aquaporin 1 | 0.3966 |
| Arhgap23 | Rho GTPase activating protein 23 | 0.4489 |
| Asb17os | ankyrin repeat and SOCS box-containing 17, opposite strand | 0.3063 |
| Asf1b | anti-silencing function 1B histone chaperone | 0.4888 |
| Asns | asparagine synthetase | 0.3843 |
| Ass1 | argininosuccinate synthetase 1 | 4.8209 |
| Atp1b2 | ATPase, Na+/K+ transporting, beta 2 polypeptide | 0.3473 |
| BC030867 | cDNA sequence BC030867 | 0.4950 |
| Bex4 | brain expressed X-linked 4 | 0.3820 |
| Bloodlinc | Bloodlinc, erythroid developmental long intergenic non-protein coding transcript | 0.2112 |
| Btnl10 | butyrophilin-like 10 | 0.4158 |
| C3 | complement component 3 | 2.0419 |
| C8g | complement component 8, gamma polypeptide | 0.4679 |
| Cacnb1 | calcium channel, voltage-dependent, beta 1 subunit | 2.0657 |
| Camp | cathelicidin antimicrobial peptide | 4.5442 |
| Ccl25 | chemokine (C-C motif) ligand 25 | 2.6092 |
| Ccna2 | cyclin A2 | 0.4398 |
| Cd177 | CD177 antigen | 3.1249 |
| Cdc25c | cell division cycle 25C | 0.3603 |
| Cdc45 | cell division cycle 45 | 0.4163 |
| Cdca3 | cell division cycle associated 3 | 0.4385 |
| Cdk1 | cyclin-dependent kinase 1 | 0.4336 |
| Cdk5rap1 | CDK5 regulatory subunit associated protein 1 | 2.1716 |
| Cdkn2c | cyclin dependent kinase inhibitor 2C | 0.4792 |
| Cebpe | CCAAT/enhancer binding protein (C/EBP), epsilon | 2.5988 |
| Cenpf | centromere protein F | 0.4365 |
| Cenph | centromere protein H | 0.3403 |
| Cep70 | centrosomal protein 70 | 0.4120 |
| Ces2g | carboxylesterase 2G | 0.3787 |
| Cfap74 | cilia and flagella associated protein 74 | 0.4673 |
| Cfd | complement factor D (adipsin) | 2.4503 |
| Chad | chondroadherin | 0.3267 |
| Chil3 | chitinase-like 3 | 2.1443 |
| Cit | citron | 0.4888 |
| Cldn13 | claudin 13 | 0.3675 |
| Clec4a2 | C-type lectin domain family 4, member a2 | 2.5024 |
| Cox20 | cytochrome c oxidase assembly protein 20 | 3.7487 |
| Cplx2 | complexin 2 | 2.5968 |
| Dapk1 | death associated protein kinase 1 | 0.4433 |
| Depdc1b | DEP domain containing 1B | 0.4797 |
| Dhrs13 | dehydrogenase/reductase (SDR family) member 13 | 0.4287 |
| Diaph3 | diaphanous related formin 3 | 0.4456 |
| Dlgap5 | DLG associated protein 5 | 0.4632 |
| Dmtn | dematin actin binding protein | 0.4898 |
| Dnph1 | 2'-deoxynucleoside 5'-phosphate N-hydrolase 1 | 0.3403 |
| Ear1 | eosinophil-associated, ribonuclease A family, member 1 | 5.0891 |
| Ear2 | eosinophil-associated, ribonuclease A family, member 2 | 2.8208 |
| Eid2 | EP300 interacting inhibitor of differentiation 2 | 0.4805 |
| Elp6 | elongator acetyltransferase complex subunit 6 | 2.0419 |
| Epdr1 | ependymin related protein 1 (zebrafish) | 0.4873 |
| Epor | erythropoietin receptor | 0.4110 |
| Ermap | erythroblast membrane-associated protein | 0.4364 |
| Fam109b | ? | 0.3690 |
| Fam219aos | family with sequence similarity 219, member A, opposite strand | 2.0419 |
| Fam24a | family with sequence similarity 24, member A | 0.2552 |
| Fbxo17 | F-box protein 17 | 3.1764 |
| Fcnb | ficolin B | 3.2898 |
| Fdx1l | ferredoxin 1-like | 2.0923 |
| Fhl1 | four and a half LIM domains 1 | 0.4051 |
| Fkbp11 | FK506 binding protein 11 | 0.4740 |
| Fn3k | fructosamine 3 kinase | 0.4522 |
| Fnip2 | folliculin interacting protein 2 | 0.4229 |
| Fpr2 | formyl peptide receptor 2 | 2.3993 |
| Gas7 | growth arrest specific 7 | 2.0140 |
| Gata1 | GATA binding protein 1 | 0.3791 |
| Gata2 | GATA binding protein 2 | 0.2978 |
| Gchfr | GTP cyclohydrolase I feedback regulator | 0.3829 |
| Gfi1b | growth factor independent 1B | 0.3735 |
| Gm10012 | cytochrome c oxidase, subunit VIIc pseudogene | 2.0905 |
| Gm12260 | predicted gene 12260 | 2.2461 |
| Gm15569 | predicted gene 15569 | 2.2461 |
| Gm15915 | predicted gene 15915 | 0.3781 |
| Gm17484 | predicted gene, 17484 | 2.7284 |
| Gm1943 | WD repeat domain 70 pseudogene | 2.6866 |
| Gm2447 | predicted gene 2447 | 2.3204 |
| Gm31108 | predicted gene, 31108 | 2.0419 |
| Gm33619 | predicted gene, 33619 | 2.0419 |
| Gm38426 | predicted gene, 38426 | 0.4634 |
| Gm38431 | predicted gene, 38431 | 0.3248 |
| Gm5483 | predicted gene 5483 | 0.2784 |
| Gm5741 | predicted gene 5741 | 0.2917 |
| Gm7443 | ribosomal protein L27 pseudogene | 0.3403 |
| Gm867 | predicted gene 867 | 0.3812 |
| Gm8817 | predicted gene 8817 | 2.0419 |
| Gm9054 | predicted gene 9054 | 2.2972 |
| Gm9828 | predicted gene 9828 | 0.3927 |
| Gpr31b | G protein-coupled receptor 31, D17Leh66b region | 0.4641 |
| Gstm5 | glutathione S-transferase, mu 5 | 0.4691 |
| H2al1k | H2A histone family member L1K | 0.4765 |
| Hdac11 | histone deacetylase 11 | 2.0828 |
| Hemgn | hemogen | 0.3807 |
| Hist1h2af | histone cluster 1, H2af | 0.4245 |
| Hist1h2ah | histone cluster 1, H2ah | 0.4264 |
| Hist1h3b | histone cluster 1, H3b | 0.4992 |
| Hist1h3c | histone cluster 1, H3c | 0.47778 |
| Hist1h3g | histone cluster 1, H3g | 0.4545 |
| Hist1h4f | histone cluster 1, H4f | 0.4313 |
| Hist1h4k | histone cluster 1, H4k | 0.4990 |
| Hk1os | hexokinase 1, opposite strand | 0.3063 |
| Hk3 | hexokinase 3 | 2.0249 |
| Hspb11 | heat shock protein family B (small), member 11 | 2.2121 |
| Hus1b | HUS1 checkpoint clamp component B | 2.1878 |
| Ifi211 | interferon activated gene 211 | 2.2428 |
| Ifitm1 | interferon induced transmembrane protein 1 | 0.4901 |
| Il13ra1 | interleukin 13 receptor, alpha 1 | 0.4986 |
| Kcnmb4 | potassium large conductance calcium-activated channel, subfamily M, beta member 4 | 0.2879 |
| Kif18b | kinesin family member 18B | 0.3358 |
| Kif22 | kinesin family member 22 | 0.4899 |
| Kif4 | kinesin family member 4 | 0.4077 |
| Klf1 | Kruppel-like factor 1 (erythroid) | 0.3478 |
| Klra13-ps | killer cell lectin-like receptor subfamily A, member 13, pseudogene | 0.4833 |
| Lamb3 | laminin, beta 3 | 2.0419 |
| Lcn2 | lipocalin 2 | 3.1374 |
| Lenep | lens epithelial protein | 2.0419 |
| Lgals4 | lectin, galactose binding, soluble 4 | 3.1024 |
| Lin7b | lin-7 homolog B (C. elegans) | 0.4538 |
| Lockd | lncRNA downstream of Cdkn1b | 0.3857 |
| Ltf | lactotransferrin | 5.1567 |
| Ly6g | lymphocyte antigen 6 complex, locus G | 3.3771 |
| Ly6g5b | lymphocyte antigen 6 complex, locus G5B | 2.7712 |
| M1ap | meiosis 1 associated protein | 0.4160 |
| Mageb16 | melanoma antigen family B, 16 | 0.4538 |
| Magef1 | melanoma antigen family F, 1 | 2.1185 |
| Mcpt8 | mast cell protease 8 | 6.1258 |
| Mest | mesoderm specific transcript | 0.4255 |
| Mfsd2b | major facilitator superfamily domain containing 2B | 0.4743 |
| Mgst3 | microsomal glutathione S-transferase 3 | 0.3769 |
| Micall2 | MICAL-like 2 | 0.2580 |
| Mmp23 | matrix metallopeptidase 23 | 2.4248 |
| Mogat1 | monoacylglycerol O-acyltransferase 1 | 2.4795 |
| Mpl | myeloproliferative leukemia virus oncogene | 0.4731 |
| Mpp2 | membrane protein, palmitoylated 2 (MAGUK p55 subfamily member 2) | 0.4339 |
| Mss51 | MSS51 mitochondrial translational activator | 0.4439 |
| Muc13 | mucin 13, epithelial transmembrane | 0.3781 |
| Mxra8 | matrix-remodelling associated 8 | 2.3422 |
| Mybl2 | myeloblastosis oncogene-like 2 | 0.4389 |
| Myl9 | myosin, light polypeptide 9, regulatory | 0.4409 |
| Ncr1 | natural cytotoxicity triggering receptor 1 | 0.4862 |
| Ndufb4 | NADH:ubiquinone oxidoreductase subunit B4 | 4.2336 |
| Nek2 | NIMA (never in mitosis gene a)-related expressed kinase 2 | 0.4948 |
| Ngp | neutrophilic granule protein | 4.1362 |
| Nhlrc4 | NHL repeat containing 4 | 0.4439 |
| Ninl | ninein-like | 0.4892 |
| Nmrk2 | nicotinamide riboside kinase 2 | 2.0419 |
| Npb | neuropeptide B | 3.0629 |
| Nrgn | neurogranin | 0.4238 |
| Nyx | nyctalopin | 0.4661 |
| P2ry1 | purinergic receptor P2Y, G-protein coupled 1 | 0.4167 |
| Parpbp | PARP1 binding protein | 0.3874 |
| Parvb | parvin, beta | 0.4901 |
| Pbk | PDZ binding kinase | 0.4459 |
| Pcbd1 | pterin 4 alpha carbinolamine dehydratase/dimerization cofactor of hepatocyte nuclear factor 1 alpha (TCF1) 1 | 0.4467 |
| Pdcd5 | programmed cell death 5 | 0.4836 |
| Pf4 | platelet factor 4 | 0.3534 |
| Pgpep1l | pyroglutamyl-peptidase I-like | 0.4492 |
| Phyhip | phytanoyl-CoA hydroxylase interacting protein | 0.4977 |
| Pkhd1l1 | polycystic kidney and hepatic disease 1-like 1 | 0.3831 |
| Pla2g4a | phospholipase A2, group IVA (cytosolic, calcium-dependent) | 0.4504 |
| Plek2 | pleckstrin 2 | 0.2955 |
| Plk1 | polo like kinase 1 | 0.4547 |
| Pltp | phospholipid transfer protein | 2.0850 |
| Plxdc1 | plexin domain containing 1 | 2.0419 |
| Pnp2 | purine-nucleoside phosphorylase 2 | 0.4863 |
| Pomc | pro-opiomelanocortin-alpha | 2.3823 |
| Prc1 | protein regulator of cytokinesis 1 | 0.4997 |
| Prg2 | proteoglycan 2, bone marrow | 14.7311 |
| Prr11 | proline rich 11 | 0.4783 |
| Prrg2 | proline-rich Gla (G-carboxyglutamic acid) polypeptide 2 | 0.3995 |
| Prss34 | protease, serine 34 | 4.0110 |
| Prtn3 | proteinase 3 | 0.4686 |
| Ptdss2 | phosphatidylserine synthase 2 | 0.4925 |
| Pusl1 | pseudouridylate synthase-like 1 | 0.4832 |
| Rab26os | RAB26, member RAS oncogene family, opposite strand | 0.4712 |
| Rag1 | recombination activating gene 1 | 2.3337 |
| Rangrf | RAN guanine nucleotide release factor | 0.2917 |
| Redrum | Redrum, erythroid developmental long intergenic non-protein coding transcript | 0.4217 |
| Rhag | Rhesus blood group-associated A glycoprotein | 0.4877 |
| Rhd | Rh blood group, D antigen | 0.3832 |
| Rnls | renalase, FAD-dependent amine oxidase | 2.4728 |
| Rpl34 | ribosomal protein L34 | 0.4810 |
| Rprl3 | ribonuclease P RNA-like 3 | 4.0840 |
| Rrm2 | ribonucleotide reductase M2 | 0.4207 |
| S100a9 | S100 calcium binding protein A9 (calgranulin B) | 2.0663 |
| Samd14 | sterile alpha motif domain containing 14 | 0.3885 |
| Sap25 | sin3 associated polypeptide | 2.4496 |
| Sapcd1 | suppressor APC domain containing 1 | 2.4557 |
| Sapcd2 | suppressor APC domain containing 2 | 0.4959 |
| Sco2 | SCO2 cytochrome c oxidase assembly protein | 2.3120 |
| Sh3tc2 | SH3 domain and tetratricopeptide repeats 2 | 0.3614 |
| Sirpb1b | signal-regulatory protein beta 1B | 0.4386 |
| Ska2 | spindle and kinetochore associated complex subunit 2 | 0.4755 |
| Slamf9 | SLAM family member 9 | 2.7942 |
| Slc22a3 | solute carrier family 22 (organic cation transporter), member 3 | 0.4558 |
| Slc25a21 | solute carrier family 25 (mitochondrial oxodicarboxylate carrier), member 21 | 0.4246 |
| Slc25a42 | solute carrier family 25, member 42 | 0.4513 |
| Slc2a4 | solute carrier family 2 (facilitated glucose transporter), member 4 | 0.2702 |
| Slc35d3 | solute carrier family 35, member D3 | 0.4281 |
| Slc6a20a | solute carrier family 6 (neurotransmitter transporter), member 20A | 0.2783 |
| Slc6a4 | solute carrier family 6 (neurotransmitter transporter, serotonin), member 4 | 0.4254 |
| Slc6a9 | solute carrier family 6 (neurotransmitter transporter, glycine), member 9 | 0.4456 |
| Spag5 | sperm associated antigen 5 | 0.4442 |
| Spata7 | spermatogenesis associated 7 | 0.4901 |
| Spdya | speedy/RINGO cell cycle regulator family, member A | 2.2062 |
| Svip | small VCP/p97-interacting protein | 0.3970 |
| Tal1 | T cell acute lymphocytic leukemia 1 | 0.3869 |
| Tctex1d4 | Tctex1 domain containing 4 | 2.0319 |
| Thyn1 | thymocyte nuclear protein 1 | 0.4968 |
| Tinagl1 | tubulointerstitial nephritis antigen-like 1 | 0.4307 |
| Tmem56 | transmembrane protein 56 | 0.4320 |
| Tmsb15b1 | thymosin beta 15b1 | 2.1020 |
| Tom1l1 | target of myb1-like 1 (chicken) | 0.3711 |
| Trem3 | triggering receptor expressed on myeloid cells 3 | 2.1205 |
| Trib3 | tribbles pseudokinase 3 | 0.4254 |
| Ttk | Ttk protein kinase | 0.4753 |
| Tyms | thymidylate synthase | 0.3979 |
| Vangl1 | VANGL planar cell polarity 1 | 0.3980 |
| Vpreb1 | pre-B lymphocyte gene 1 | 2.8077 |
| Wfdc21 | WAP four-disulfide core domain 21 | 2.4390 |
| Xlr3b | X-linked lymphocyte-regulated 3B | 0.3232 |
| Zfp11 | zinc finger protein 11 | 2.1440 |
| Zfp459 | zinc finger protein 459 | 2.1454 |

**Supplementary** **Table S3.** The list of non-coding RNA genes in total splenocytes with significant changes (expression ration IDO shRNA / Scr IDO shRNA >2 or < 0.5, RPKM > 0.1).

| **Official gene symbol** | **Name** | **Expression ratio** |
| --- | --- | --- |
| 1110019D14Rik | RIKEN cDNA 1110019D14 gene(1110019D14Rik) | 2.0061 |
| 1700001D01Rik | RIKEN cDNA 1700001D01 gene(1700001D01Rik) | 2.0479 |
| 1700019D03Rik | RIKEN cDNA 1700019D03 gene(1700019D03Rik) | 0.4388 |
| 1700065D16Rik | RIKEN cDNA 1700065D16 gene(1700065D16Rik) | 0.4922 |
| 1700084E18Rik | RIKEN cDNA 1700084E18 gene(1700084E18Rik) | 0.3192 |
| 1700092M07Rik | RIKEN cDNA 1700092M07 gene(1700092M07Rik) | 0.2633 |
| 1700100L14Rik | RIKEN cDNA 1700100L14 gene(1700100L14Rik) | 0.4388 |
| 2010310C07Rik | RIKEN cDNA 2010310C07 gene(2010310C07Rik) | 0.4937 |
| 2310010J17Rik | RIKEN cDNA 2310010J17 gene(2310010J17Rik) | 0.4388 |
| 4930474N09Rik | RIKEN cDNA 4930474N09 gene(4930474N09Rik) | 0.4157 |
| 4930519L02Rik | RIKEN cDNA 4930519L02 gene(4930519L02Rik) | 2.0479 |
| 4930522P08Rik | RIKEN cDNA 4930522P08 gene(4930522P08Rik) | 2.1942 |
| 4930562C15Rik | RIKEN cDNA 4930562C15 gene(4930562C15Rik) | 0.4911 |
| 4930564C03Rik | RIKEN cDNA 4930564C03 gene(4930564C03Rik) | 0.4388 |
| 4933431G14Rik | RIKEN cDNA 4933431G14 gene(4933431G14Rik) | 2.7427 |
| 4933439K11Rik | RIKEN cDNA 4933439K11 gene(4933439K11Rik) | 2.2819 |
| 9330175E14Rik | RIKEN cDNA 9330175E14 gene(9330175E14Rik) | 2.2896 |
| A330009N23Rik | RIKEN cDNA A330009N23 gene(A330009N23Rik) | 0.4579 |
| A630019I02Rik | RIKEN cDNA A630019I02 gene(A630019I02Rik) | 2.0138 |
| A730085K08Rik | RIKEN cDNA A730085K08 gene(A730085K08Rik) | 2.0479 |
| A930015D03Rik | RIKEN cDNA A930015D03 gene(A930015D03Rik) | 3.5684 |
| F630028O10Rik | RIKEN cDNA F630028O10 gene(F630028O10Rik) | 0.3114 |
| B830017H08Rik | RIKEN cDNA B830017H08 gene(B830017H08Rik) | 3.0606 |
| C530008M17Rik | RIKEN cDNA C530008M17 gene(C530008M17Rik) | 0.3325 |
| D630041G03Rik | RIKEN cDNA D630041G03 gene(D630041G03Rik) | 2.4131 |
| E130218I03Rik | RIKEN cDNA E130218I03 gene(E130218I03Rik) | 3.1245 |
| Mir10a | microRNA 10a(Mir10a) | 0.2926 |
| Mir1190 | microRNA 1190(Mir1190) | 43.0055 |
| Mir143 | microRNA 143(Mir143) | 37.7396 |
| Mir148a | microRNA 148a(Mir148a) | 0.4388 |
| Mir1668 | microRNA 1668(Mir1668) | 0.4388 |
| Mir1955 | microRNA 1955(Mir1955) | 0.4388 |
| Mir207 | microRNA 207(Mir207) | 2.6330 |
| Mir215 | microRNA 215(Mir215) | 2.6330 |
| Mir29a | microRNA 29a(Mir29a) | 45.8724 |
| Mir30d | microRNA 30d(Mir30d) | 4.6809 |
| Mir466i | microRNA 466i(Mir466i) | 0.4388 |
| Mir5099 | microRNA 5099(Mir5099) | 0.3511 |
| Mir5107 | microRNA 5107(Mir5107) | 2.6330 |
| Mir5123 | microRNA 5123(Mir5123) | 0.1254 |
| Mir5126 | microRNA 5126(Mir5126) | 2.6330 |
| Mir6374 | microRNA 6374(Mir6374) | 0.2194 |
| Mir6385 | microRNA 6385(Mir6385) | 0.1121 |
| Mir6516 | microRNA 6516(Mir6516) | 2.6330 |
| Mir6537 | microRNA 6537(Mir6537) | 0.4388 |
| Mir6541 | microRNA 6541(Mir6541) | 0.0864 |
| Mir6992 | microRNA 6992(Mir6992) | 0.1463 |
| Mir7060 | microRNA 7060(Mir7060) | 0.3511 |
| Mir7063 | microRNA 7063(Mir7063) | 3.5107 |
| Mir7-1 | microRNA 7-1(Mir7-1) | 4.3883 |
| Mir8100 | microRNA 8100(Mir8100) | 0.1097 |
| Mir8102 | microRNA 8102(Mir8102) | 0.4388 |
| Mir8118 | microRNA 8118(Mir8118) | 0.4388 |
| Mirlet7f-2 | microRNA let7f-2(Mirlet7f-2) | 19.5409 |
| Mirlet7g | microRNA let7g(Mirlet7g) | 69.9864 |
| Rnu73b | U73B small nuclear RNA | 2.04789 |
| Scarna3a | small Cajal body-specific RNA 3A | 0.4646 |
| Scarna3b | small Cajal body-specific RNA 3B | 0.2926 |
| Scarna8 | small Cajal body-specific RNA 8 | 0.1881 |
| Smim1 | small integral membrane protein 1 | 3.3931 |
| Snora15 | small nucleolar RNA, H/ACA box 15 | 0.2826 |
| Snora16a | small nucleolar RNA, H/ACA box 16A | 0.4388 |
| Snora17 | small nucleolar RNA, H/ACA box 17 | 0.3462 |
| Snora21 | small nucleolar RNA, H/ACA box 21 | 0.4111 |
| Snora26 | small nucleolar RNA, H/ACA box 26 | 0.3002 |
| Snora2b | small nucleolar RNA, H/ACA box 2B | 0.4388 |
| Snora3 | small nucleolar RNA, H/ACA box 3 | 0.4909 |
| Snora30 | small nucleolar RNA, H/ACA box 30 | 0.3511 |
| Snora31 | small nucleolar RNA, H/ACA box 31 | 0.2360 |
| Snora33 | small nucleolar RNA, H/ACA box 33 | 0.4341 |
| Snora52 | small nucleolar RNA, H/ACA box 52 | 0.4376 |
| Snora5c | small nucleolar RNA, H/ACA box 5C | 0.3049 |
| Snora62 | small nucleolar RNA, H/ACA box 62 | 0.4388 |
| Snora64 | small nucleolar RNA, H/ACA box 64 | 0.2689 |
| Snora65 | small nucleolar RNA, H/ACA box 65 | 0.2592 |
| Snora68 | small nucleolar RNA, H/ACA box 68 | 0.2484 |
| Snora69 | small nucleolar RNA, H/ACA box 69 | 0.2508 |
| Snora78 | small nucleolar RNA, H/ACA box 7 | 0.3161 |
| Snord100 | small nucleolar RNA, C/D box 100 | 0.2508 |
| Snord12 | small nucleolar RNA, C/D box 12 | 0.1463 |
| Snord15a | small nucleolar RNA, C/D box 15A | 0.4135 |
| Snord16a | small nucleolar RNA, C/D box 16A | 0.4151 |
| Snord22 | small nucleolar RNA, C/D box 22 | 0.3470 |
| Snord23 | small nucleolar RNA, C/D box 23 | 0.3761 |
| Snord34 | small nucleolar RNA, C/D box 34 | 0.2926 |
| Snord35a | small nucleolar RNA, C/D box 35A | 0.3343 |
| Snord49b | small nucleolar RNA, C/D box 49B | 0.3191 |
| Snord55 | small nucleolar RNA, C/D box 55 | 0.1755 |
| Snord67 | small nucleolar RNA, C/D box 67 | 0.3135 |
| Snord8 | small nucleolar RNA, C/D box 8 | 0.2525 |
| Snord83b | small nucleolar RNA, C/D box 83B | 0.3761 |
| Snord89 | small nucleolar RNA, C/D box 89 | 0.3761 |

**Supplementary Table S4.** The list of non-coding RNA genes in splenic neutrophils with significant changes (expression ration IDO shRNA / Scr IDO shRNA >2 or < 0.5, RPKM > 0.1).

| **Official gene symbol** | **Name** | **Expression ratio** |
| --- | --- | --- |
| 1700020D05Rik | RIKEN cDNA 1700020D05 gene | 2.6545 |
| 1700066J03Rik | RIKEN cDNA 1700066J03 gene | 2.0419 |
| 1700120K04Rik | RIKEN cDNA 1700120K04 gene | 2.2688 |
| 1810012K16Rik | RIKEN cDNA 1810012K16 gene | 2.2972 |
| 2010109I03Rik | RIKEN cDNA 2010109I03 gene | 2.0419 |
| 2810025M15Rik | RIKEN cDNA 2810025M15 gene | 0.4899 |
| 3300002I08Rik | RIKEN cDNA 3300002I08 gene | 0.4354 |
| 4930596I21Rik | RIKEN cDNA 4930596I21 gene | 0.4765 |
| 4933407G14Rik | RIKEN cDNA 4933407G14 gene | 2.0419 |
| C530008M17Rik | RIKEN cDNA C530008M17 gene | 0.4916 |
| Mir1931 | microRNA 1931 | 0.2552 |
| Mir1949 | microRNA 1949 | 3.0629 |
| Mir1a-1hg | Mir1a-1 and Mir133a-2 host gene | 2.4181 |
| Mir27a | microRNA 27a | 2.3823 |
| Mir3066 | microRNA 3066 | 0.2042 |
| Mir5099 | microRNA 5099 | 4.0839 |
| Mir5126 | microRNA 5126 | 0.2854 |
| Mir6236 | microRNA 6236 | 0.2741 |
| Mir6374 | microRNA 6374 | 4.0839 |
| Mir6385 | microRNA 6385 | 2.0419 |
| Mir6415 | microRNA 6415 | 2.0419 |
| Mir6516 | microRNA 6516 | 0.4885 |
| Mir684-1 | microRNA 684-1 | 3.3468 |
| Mir7060 | microRNA 7060 | 2.0419 |
| Mir8094 | microRNA 8094 | 0.4527 |
| Mir8102 | microRNA 8102 | 0.4084 |
| Mir8118 | microRNA 8118 | 0.3403 |
| Scarna10 | small Cajal body-specific RNA 10 | 0.3403 |
| Scarna8 | small Cajal body-specific RNA 8 | 0.4850 |
| Snora24 | small nucleolar RNA, H/ACA box 24 | 0.3139 |
| Snora31 | small nucleolar RNA, H/ACA box 31 | 0.4547 |
| Snora47 | small nucleolar RNA, H/ACA box 47 | 0.4850 |
| Snora75 | small nucleolar RNA, H/ACA box 75 | 0.3403 |
| Snord100 | small nucleolar RNA, C/D box 100 | 4.0839 |
| Snord11 | small nucleolar RNA, C/D box 11 | 0.3403 |
| Snord2 | small nucleolar RNA, C/D box 2 | 0.1459 |
| Snord49a | small nucleolar RNA, C/D box 49A | 2.0419 |
| Snord58b | small nucleolar RNA, C/D box 58B | 0.3403 |
| Snord61 | small nucleolar RNA, C/D box 61 | 0.3403 |
| Snord65 | small nucleolar RNA, C/D box 65 | 2.0419 |
| Snord83b | small nucleolar RNA, C/D box 83B | 0.4148 |
| Snord87 | small nucleolar RNA, C/D box 87 | 3.0629 |
